# Defining the Identity and Dynamics of Adult Gastric Isthmus Stem Cells

**DOI:** 10.1016/j.stem.2019.07.008

**Published:** 2019-09-05

**Authors:** Seungmin Han, Juergen Fink, David J. Jörg, Eunmin Lee, Min Kyu Yum, Lemonia Chatzeli, Sebastian R. Merker, Manon Josserand, Teodora Trendafilova, Amanda Andersson-Rolf, Catherine Dabrowska, Hyunki Kim, Ronald Naumann, Ji-Hyun Lee, Nobuo Sasaki, Richard Lester Mort, Onur Basak, Hans Clevers, Daniel E. Stange, Anna Philpott, Jong Kyoung Kim, Benjamin D. Simons, Bon-Kyoung Koo

**Affiliations:** 1 Wellcome Trust–Medical Research Council Stem Cell Institute, University of Cambridge, Cambridge CB2 1QR, UK; 2 The Wellcome Trust/Cancer Research UK Gurdon Institute, University of Cambridge, Cambridge CB2 1QN, UK; 3 Department of Genetics, University of Cambridge, Cambridge CB2 3EH, UK; 4 Department of Applied Mathematics and Theoretical Physics, Centre for Mathematical Sciences, University of Cambridge, Wilberforce Road, Cambridge CB3 0WA, UK; 5 Department of New Biology, DGIST, Daegu 42988, Republic of Korea; 6 Department of Visceral, Thoracic and Vascular Surgery, University Hospital Carl Gustav Carus, Medical Faculty, Technische Universität Dresden, Fetscherstr. 74, 01307 Dresden, Germany; 7 MPI of Molecular Cell Biology and Genetics, Pfotenhauerstrasse 108, 01307 Dresden, Germany; 8 Institute of Molecular Biotechnology of the Austrian Academy of Sciences (IMBA), Vienna Biocenter (VBC), Dr. Bohr-Gasse 3, 1030 Vienna, Austria; 9 Division of Gastroenterology and Hepatology, Department of Internal Medicine, Keio University School of Medicine, 35 Shinanomachi, Shinjuku-ku, Tokyo 160-8582, Japan; 10 Division of Biomedical and Life Sciences, Faculty of Health and Medicine, Furness Building, Lancaster University, Bailrigg, Lancaster LA1 4YG, UK; 11 Hubrecht Institute, Royal Netherlands Academy of Arts and Sciences (KNAW) and University Medical Center Utrecht, 3584 Utrecht, the Netherlands; 12 Department of Oncology, Hutchison/MRC Research Centre, University of Cambridge, Cambridge Biomedical Campus, Cambridge CB2 0XZ, UK

**Keywords:** gastric corpus isthmus stem cell, two stem cell compartments, punctuated neutral drift, unbiased genetic labeling, deep tissue imaging, biophysical modeling, single-cell RNA-seq, Lgr5, Troy, intestine

## Abstract

The gastric corpus epithelium is the thickest part of the gastrointestinal tract and is rapidly turned over. Several markers have been proposed for gastric corpus stem cells in both isthmus and base regions. However, the identity of isthmus stem cells (IsthSCs) and the interaction between distinct stem cell populations is still under debate. Here, based on unbiased genetic labeling and biophysical modeling, we show that corpus glands are compartmentalized into two independent zones, with slow-cycling stem cells maintaining the base and actively cycling stem cells maintaining the pit-isthmus-neck region through a process of “punctuated” neutral drift dynamics. Independent lineage tracing based on Stmn1 and Ki67 expression confirmed that rapidly cycling IsthSCs maintain the pit-isthmus-neck region. Finally, single-cell RNA sequencing (RNA-seq) analysis is used to define the molecular identity and lineage relationship of a single, cycling, IsthSC population. These observations define the identity and functional behavior of IsthSCs.

## Highlights

• Marker-free lineage tracing reveals two types of stem cells in the gastric corpus
• Actively cycling isthmus stem cells follow “punctuated” neutral drift dynamics
• Stmn1 and Ki67 lineage tracing confirms the active cycling of isthmus stem cells
• Single-cell RNA-seq defines identity and lineage relationship of isthmus stem cells

## Graphical Abstract

**Figure:**
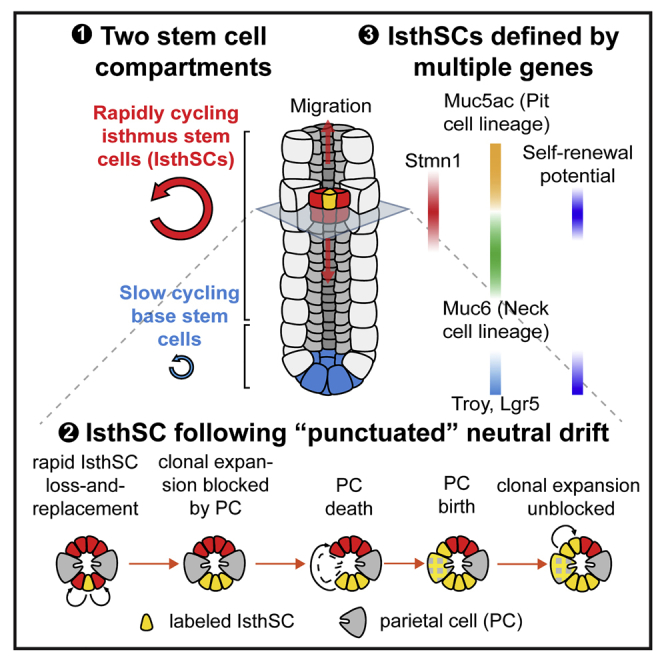

## Teaser

Using lineage-tracing assays and single-cell gene expression profiling, Han et al. show that the mouse gastric corpus gland is compartmentalized, with base and isthmus regions supported by two separate independent stem cell populations. Isthmus stem cells are rapidly cycling, with a broad expression signature, and undergo “punctuated” neutral drift dynamics.

## Introduction

The gastric corpus epithelium consists of long, single-layered glands populated by diverse gastric cell types, including pit, neck, parietal, and chief cells (Bartfeld and Koo, 2017, Mills and Shivdasani, 2011, Willet and Mills, 2016, Kim and Shivdasani, 2016). Pit cells are located close to the stomach lumen and adjacent to proliferating cells in the isthmus region. Mucus neck and chief cells are located below the isthmus, toward the gland base. Acid-secreting parietal cells are present throughout the gland, except for the pit region. In common with other gastrointestinal (GI) epithelia, the corpus epithelium is replenished rapidly and constantly.

As with other components of the GI tract, the corpus epithelium hosts a stem cell population in the gland base, marked by canonical, Wnt-responsive, stem cell markers, such as Troy and Lgr5. However, these cells, which we term “base stem cells (BSCs),” have characteristics distinct from stem cells in other GI tract components (Leushacke et al., 2017, Stange et al., 2013): during homeostasis, BSCs are largely quiescent and rarely contribute to the turnover of the epithelium. However, upon injury, BSCs proliferate rapidly and replenish the whole gland, the hallmark of a reserve stem cell population (Leushacke et al., 2017, Stange et al., 2013).

In contrast to other regions of the GI tract, the corpus epithelium exhibits a unique architecture, with a domain of rapidly cycling progenitors in the upper-middle part of the gland. Based on radiolabeling and electron microscopy (Karam and Leblond, 1993a, Karam and Leblond, 1993b, Karam and Leblond, 1993c), and later using transgene mutation (Bjerknes and Cheng, 2002), it was argued that this region must play host to a long-term, constantly proliferating isthmus stem cell (IsthSC) population (Sáenz and Mills, 2018). However, this hypothesis has not been validated rigorously (Willet and Mills, 2016). Based on lineage-tracing approaches, several stem cell marker genes have been proposed, including Sox2, Runx1, Lrig1, Mist1 (Bhlha15), and Bmi1 (Arnold et al., 2011, Choi et al., 2018, Hayakawa et al., 2015, Matsuo et al., 2017, Yoshioka et al., 2019). However, these markers have proven insufficient to define IsthSC identity and function. First, these markers are not region specific, with expression found in cells present in both isthmus and base regions, making the origin of long-lived clones in genetic tracing models ambiguous. Second, the various putative stem cell markers are expressed by different cells in the isthmus region with distinct characteristics. For example, Sox2 and Mist1 appear to mark quiescent populations but are not co-expressed (Hayakawa et al., 2015). Runx1+ isthmus cells are positive for the proliferation marker Ki67, and Mist1+ isthmus cells are Ki67− (Matsuo et al., 2017). It is also unclear which of the aforementioned markers correlate with Lrig1+ isthmus cells (Choi et al., 2018). Importantly, it remains uncertain as to whether these markers are co-expressed by individual cell types or mark different corpus cell types. Due to these uncertainties, the existence, identity, multiplicity, and potency of IsthSCs are widely considered to be unresolved. Indeed, it is still not clear whether IsthSCs are quiescent or active or whether they constitute a long-lived population or a transient descendent of BSCs.

To define the identity and function of IsthSCs, we used an unbiased, marker-free, lineage-tracing approach, combining ubiquitous labeling with the multi-color confetti mouse reporter system (Snippert et al., 2010). Our findings reveal that there are two distinct stem cell populations governing the upper and lower parts of gastric corpus glands. Analysis of the clonal dynamics reveals a process of “punctuated” neutral drift in the isthmus region, showing that actively cycling IsthSCs serve as the main source of turnover. Using lineage-tracing strategies based on the expression of the proliferation markers Stmn1 and Ki67, we confirmed the actively cycling nature of the IsthSC pool. Finally, we used single-cell RNA sequencing (scRNA-seq) analysis to define the molecular heterogeneity and lineage relationship of isthmus cells, confirming a broad expression signature of IsthSC marker genes. These findings show that rapidly cycling cells in the isthmus region function as the definitive IsthSC population governing the turnover of the pit-isthmus-neck region of the corpus gland.

## Results

### Stomach Corpus Glands Are Maintained by Two Independent Stem Cell Populations

**Figure 1:**
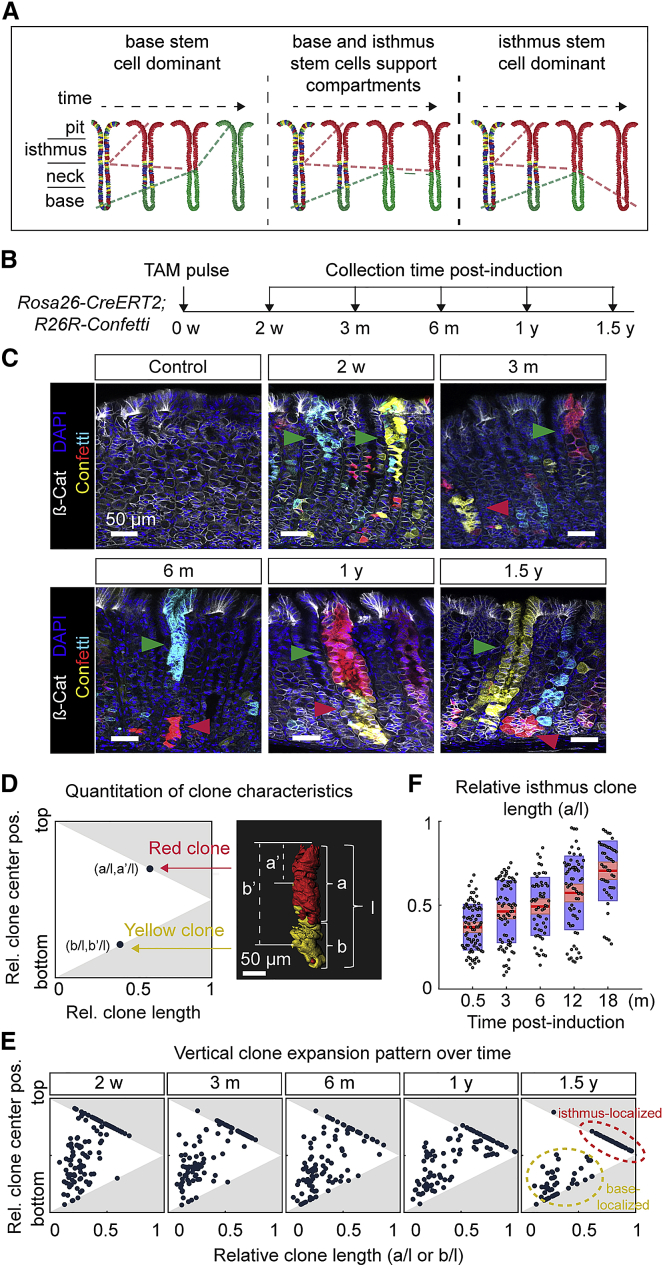
Stomach Corpus Glands Are Maintained by Two Independent Stem Cell Populations (A) Schematic illustrating potential outcome of clonal expansion in stomach corpus glands based on the long-term contribution of isthmus and base stem cells. Left panel: slowly cycling base stem cells replace isthmus progenitors over time. Middle panel: isthmus and base stem cells maintain their respective compartments over time. Right panel: IsthSCs replace base stem cells over time. (B) Experimental schedule for tracing. (C) Representative images from 150-μm-thick z stack confocal images of stomach corpus of *Rosa26-CreERT2;R26R-Confetti* mice at 2 weeks, 3 months, 6 months, 1 year, and 1.5 years following TAM injection. Isthmus- and base-localized clones are indicated by green and red arrowheads, respectively. Yellow, EYFP; red, tdimer2; cyan, mCerulean; grey, β-catenin; blue, DAPI. Scale bars: 50 μm. (D) Schematic illustrating the quantification of clone characteristics based on the midpoint and length. Scale bar: 50 μm. (E) Scatterplot of relative (vertical) clone length and center position in *Rosa26-CreERT2;R26R-Confetti* mice. Clone characteristics illustrate a separation over time into larger clones (inside red dotted ellipse) located in the isthmus-pit region and smaller clones (inside yellow dotted ellipse) located in the base region of glands. N = 114 clones (2 weeks), 109 clones (3 months), 104 clones (6 months), 99 clones (1 year), and 82 clones (1.5 years) were pooled from 2 mice per time point. (F) Distribution of the relative length of labeled clones in the isthmus region to gland length over time based on *Rosa26-CreERT2;R26R-Confetti* tracing. Dots denote lengths of individual clones (N = 77 clones [2 weeks], 71 clones [3 months], 56 clones [6 months], 67 clones [1 year], and 48 clones [1.5 years]) pooled from 2 mice per time point. Red line, mean; red-shaded box, 95% confidence interval (CI); blue-shaded box, SD. See Figures S1 and S2.

Based on the current literature, clonogenic activity is expected to occur in two regions of the gastric corpus gland: one is located in the (proliferatively active) isthmus region and the other is in the gland base (Phesse and Sansom, 2017, Willet and Mills, 2016). However, the potential lineage relationship, potency, and function of these populations is still debated (Figure 1A). To probe the long-term fate of isthmus cells, we sought to define the clonal dynamics of individual lineages in corpus glands using the 4-color (green, yellow, red, and cyan) *Rosa26-StopFlox-Confetti* (*R26R-Confetti*) reporter system and a ubiquitously expressed *CreER* allele. Using deep-tissue imaging (Figure S1), together with nuclear (DAPI) and plasma membrane staining (β-catenin), we resolved clones at single-cell resolution throughout the entire corpus region over a 3-month time course. Among various ubiquitous, inducible Cre lines, we settled on the *Rosa26-CreERT2;R26R-Confetti* mouse as optimal (Figure S2). We then performed long-term lineage tracing using the *Rosa26-CreERT2;R26R-Confetti* mouse up to 1.5 years (Figures 1B and 1C).

At 2 weeks post-tamoxifen (TAM) administration, large isthmus-localized clones were readily identifiable, and smaller clones lay scattered throughout the base (Figure 1C). After 3 months, base clones had begun to expand, while remaining base localized (Figure 1C, red arrowheads), and isthmus-spanning clones were restricted to the upper part of the gland (Figure 1C, green arrowheads). This pattern of behavior—isthmus-spanning upper clones and base-localized small clones—prevailed over the entire time course. Charting the length and center of clones (Figure 1D), we found evidence for their progressive segregation into two types (Figure 1E). On average, an isthmus-spanning clone occupied some 80% (±SD20%) of the whole gland in the upper region, and a base-localized clone occupied some 30% (±SD10%) at 18 months post-induction (Figures S2G and S2H), supporting a scenario in which individual corpus glands are maintained by two equally long-lived but distinct stem cell compartments (Figure 1A, center).

At high doses, TAM can lead to damage of parietal cells (Huh et al., 2012) that, in turn, can cause proliferation in the base (Leushacke et al., 2017); in this context, the vertical lengths of base clones were relatively high as compared to those reported by Leushacke et al. (2017), most likely due to the higher dose of TAM (3 mg/20 g mouse body weight). However, notably, in both our current study and that of Leushacke et al., after an initial burst of proliferation in the base following induction, base clones show the same low degree of expansion over the long term (Figure S2G; cf. the fast vertical expansion of upper clones, Figure S2H). This suggests that the early expansion represents a transient, damage-mediated response to TAM. However, the limited extent of expansion, and its subsequent arrest, suggests that marked cells at the gland base do not make a long-term contribution to the isthmus region, supporting the inferred compartmentalization of tissue. To reinforce this conclusion, we also examined clonal organization in the *Rosa26-rtTA;tetO-Cre;R26R-Confetti* system. Notably, based on this doxycycline-mediated labeling strategy, traced over 3 months, the pattern of tracing was almost identical to that of the *Rosa26-CreERT2;R26R-Confetti* model (Figures S2I and S2J), confirming the existence of two long-lived but distinct compartments.

Previously, it has been shown that individual clones spanning the entire gland can emerge under conditions of injury and repair (Leushacke et al., 2017, Stange et al., 2013). In the current analysis, it was notable that very few clones (∼2.4% at 18 months post-induction) occupy entire glands (Figure 1E), indicating the longevity of both compartments under homeostatic conditions.

### The Pit-Isthmus-Neck Region of the Stomach Corpus Gland Is Maintained by Stochastic Loss and Replacement of IsthSCs

**Figure 2:**
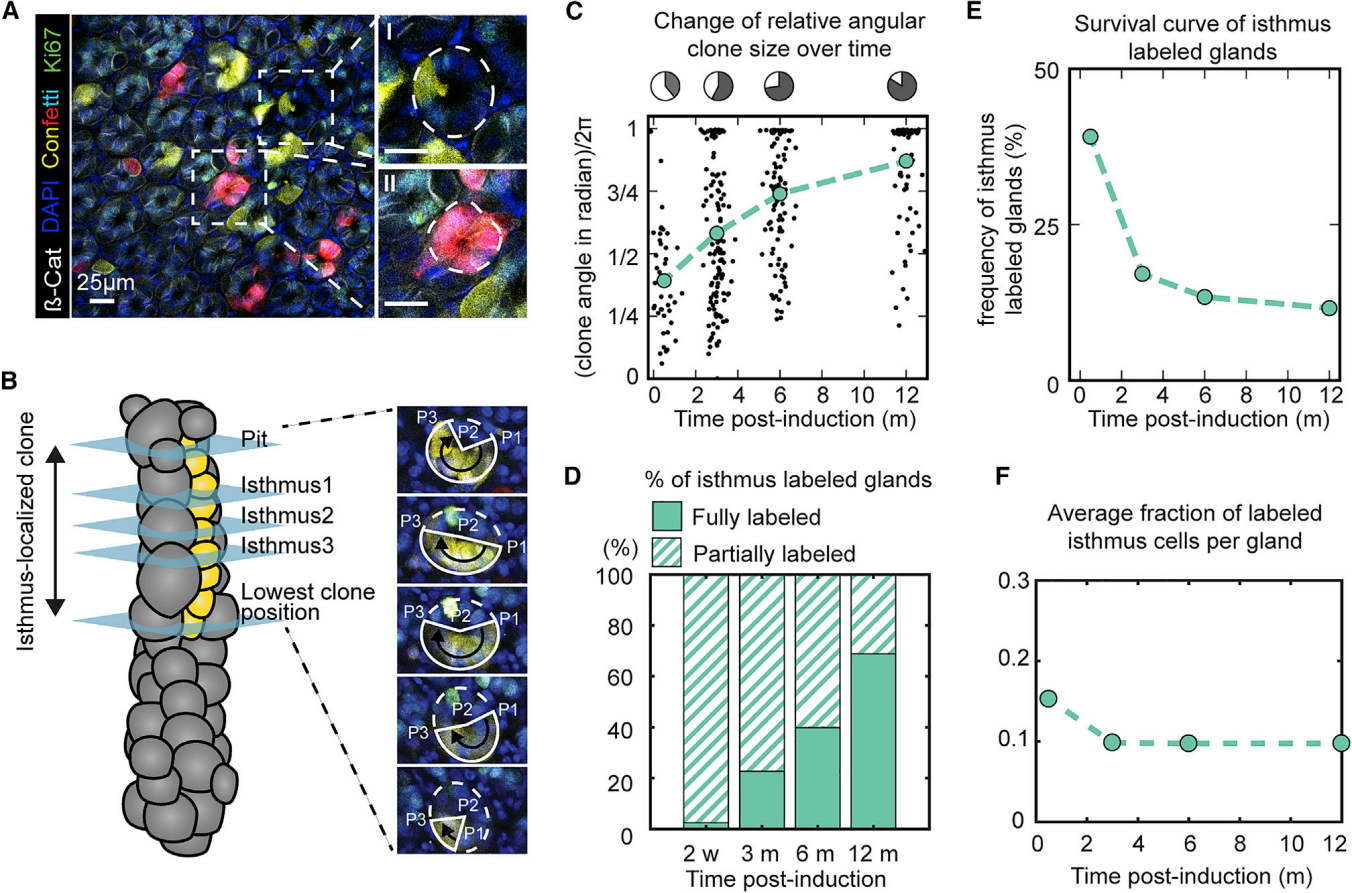
The Isthmus-Pit Region of the Stomach Corpus Gland Is Maintained by Stochastic Loss and Replacement of IsthSCs (A) Representative whole-mount image of stomach corpus from *Rosa26-CreERT2;R26R-Confetti* mice at 3 months post-induction. Ki67 antibody staining of proliferative cells (green) identifies the isthmus zone. Magnified panels to the right show examples of partially (I) and fully (II) labeled glands. Green: Ki67. Scale bars: 25 μm. (B) Scoring strategy to quantify lateral clone expansion by measuring the relative angular size of clones around the gland circumference. Relative angular size was measured in pit, isthmus, and neck regions at 3 months post-induction. Left panel: schematic shows the contribution of a yellow isthmus clone. The 3 sections depict the top (isthmus 1), center (isthmus 2), and lowest (isthmus 3) position of the isthmus zone, as defined by Ki67 labeling. Right panel: in each optical section, the clone dimensions are defined by 3 points (P1, P2, P3, and connecting white lines), which determine the angular clone size. (C) The relative angular isthmus clone size. Black dots represent individual clones and green dots the average. N = 40 clones (2 weeks), 132 clones (3 months), 103 clones (6 months), and 90 clones (12 months) were pooled from 1–3 mice per time point. (D) Percentage of fully and partially labeled glands at indicated time points. (E) Clone survival curve for isthmus-labeled glands. Green dots denote the frequency calculated from the number of glands labeled in the isthmus region relative to the total number of glands for 1–3 mice at each time point. (F) Average fraction of labeled isthmus cells (green dots) obtained as the product of the average clone size (C) and frequency (E). The dotted lines linking green dots in (C), (E), and (F) show the trend over time. See Video S1 and Figure S3.

To understand the behavior of isthmus-derived clones, we analyzed their dynamics based on their vertical and lateral expansion around the gland by performing whole-mount imaging following deep tissue labeling (STAR Methods). Previously, quantitative studies of clonal dynamics in the mouse small intestine and colon have shown that turnover is supported by the competition of intestinal stem cells (ISCs) for niche access at the crypt base, as evidenced by the “neutral drift dynamics” of stem-cell-derived clones (Lopez-Garcia et al., 2010, Snippert et al., 2010, Ritsma et al., 2014). Based on the rapid vertical expansion of isthmus-derived clones (Figures 1C and 1F) and their cohesive character, we expected that neutral competition between actively dividing IsthSCs would drive a similarly rapid lateral drift of clones around the circumference of the isthmus, mirroring the clonal dynamics of the crypt. However, over the long-term, partially labeled isthmus regions (Figure 2AI) were found to be much more abundant than fully labeled regions (Figure 2AII): when quantified by the angular span of isthmus clones (Figure 2B; Video S1), we found that it took 3 months for the average clone size to extend to more than 50% of the gland circumference. Even at 12 months, some isthmus regions remained only partially labeled (Figures 2C and 2D), suggesting that the drift of clones toward monoclonality in the isthmus region is unexpectedly slow, given the seemingly rapid rate of cell division in this region, as evidenced by the vertical clone expansion.

Video S1. The Strategy to Score Lateral Clone Size, Related to Figure 2 This movie demonstrates the scoring strategy to quantify lateral clone expansion by measuring the relative angular size of clones around the gland circumference from the whole mount image of the stomach corpus of *Rosa26-CreERT2;R26R-Confetti* mice.

To explain this behavior, we first considered whether the medium- and long-term dynamics of isthmus clones were consistent with the labeling of a renewing population. Indeed, after an initial abrupt loss of clones derived from cells with short-term proliferative potential, the steady increase in average clone size (Figure 2C) was compensated by a decrease in clone persistence (Figure 2E) so that the average fraction of labeled cells remained approximately constant over the time course (Figure 2F), i.e., by 3 months post-labeling, clones are enriched in those derived from stem cells with long-term renewal potential.

To understand the dynamics of lateral clonal expansion, we first invoked a stochastic model of stem cell fate describing lateral cell competition in the isthmus region. Our starting point was the “orthodox” model of neutral drift introduced to describe clonal dynamics in the intestinal crypt (Lopez-Garcia et al., 2010). In this model, stem cell division is correlated with the displacement of neighbors from their niche and their entry into a differentiation program, so that the size of the stem cell compartment remains constant over time. In the intestine, the localization of the stem cell compartment to the crypt base leads to a unidirectional displacement of differentiated cells, resulting in a rapid vertical expansion of clones along the crypt axis and a slower lateral expansion around the circumference. In the context of the stomach corpus, the association of the stem cell niche with the isthmus domain leads to an unusual bidirectional displacement of cells both upwards into the pit and downwards into the neck region (Figure 1A).

Within this framework, the clonal dynamics depends in principle on two parameters: the rate of IsthSC division and the frequency with which such divisions lead to stem cell loss and replacement around the gland. However, because our focus is on the slow lateral expansion of clones, inspired by studies of the intestinal crypt, we considered a simpler one-parameter model in which the IsthSC compartment is characterized by a one-dimensional chain of cells in which, at rate λ, loss through displacement and differentiation is compensated by the symmetric division of a neighbor (for details, see Methods S1). Note that such a model represents a caricature of what is likely to be a more complex organization, in which the long-term survival potential may be biased or primed by location within the niche, so that the “effective” IsthSC number, N, may underestimate the number of IsthSCs that have the potential to support long-term self-renewal (Ritsma et al., 2014).

From the measurements of average clone size, we found that the neutral drift model could provide a satisfactory fit to the data (Figure S3A) with a stem cell loss-and-replacement rate of $\lambda /N^{2}=0.03/\text{month}$. Indeed, this ratio is some four times slower than that reported in the intestinal crypt (Lopez-Garcia et al., 2010). Moreover, if we equate N with the typical number of dividing isthmus cells within a circumferential section of the gland (estimated at around 8), this ratio translates to a loss-and-replacement rate of around once per 2 weeks, a figure much lower than the expected cell cycle time of IsthSCs.

### Parietal Cells Establish a Barrier to the Lateral Expansion of Isthmus Clones

**Figure 3:**
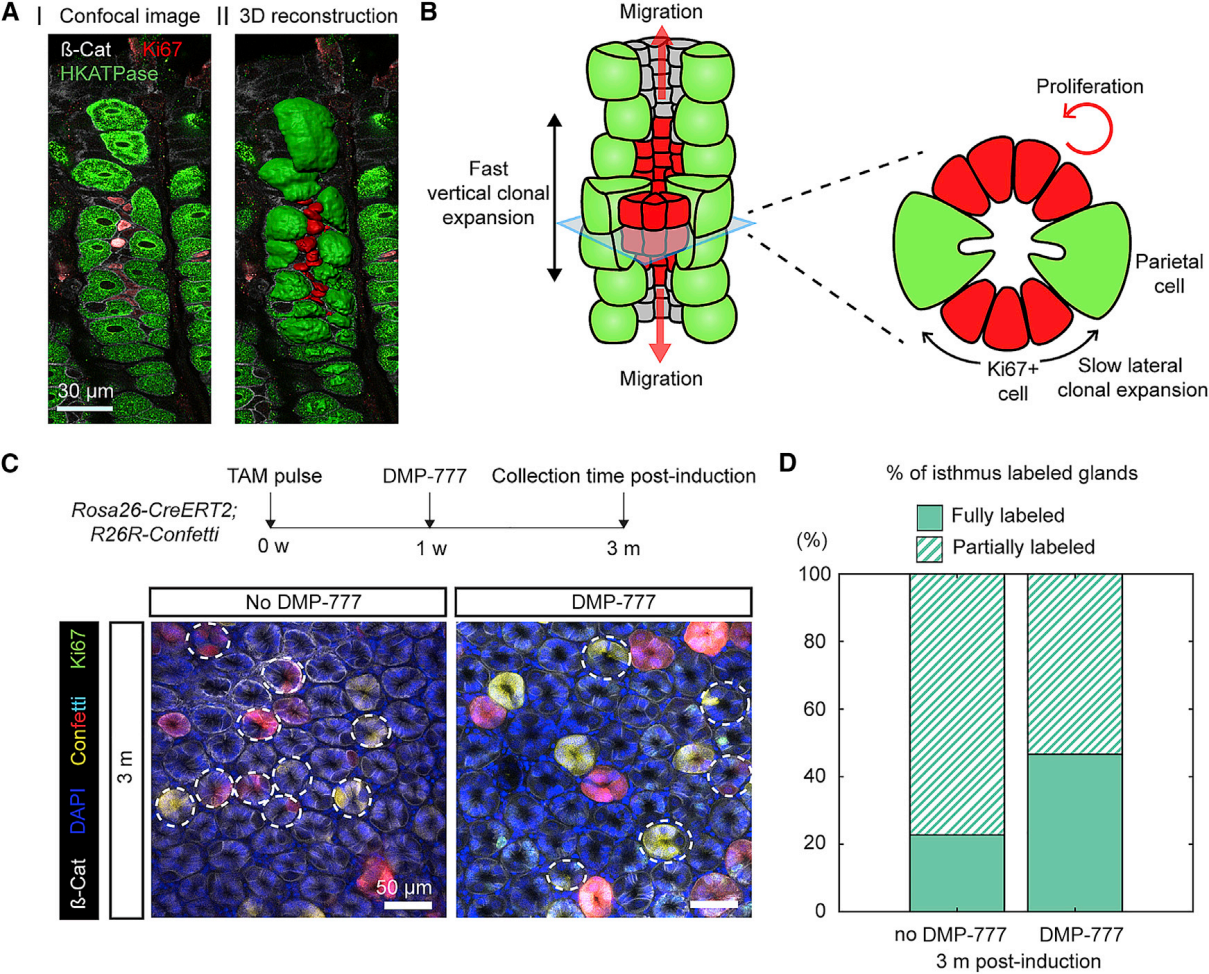
Parietal Cells Act as Physical Barriers against Lateral Clone Expansion (A) Representative confocal (63×) image of the pit-isthmus-neck region from 150-μm-thick section of mouse stomach corpus illustrating cellular organization (I). 3D reconstruction of the confocal z stack images was performed using IMARIS (Bitplane; II). Small, Ki67+ cells are tightly packed between columns of large HKATPase+ parietal cells, suggesting that the lateral expansion of Ki67+ cells may be inhibited. Red, Ki67; Green, HKATPase. (B) Schematic depicting potential role of parietal cells as barriers to lateral expansion in the isthmus region. From the gland architecture, progeny of Ki67+ proliferating cells may move vertically (parallel to the gland axis) through the “valley” formed between parietal cells, resulting in rapid vertical expansion (left diagram). By contrast, the lateral expansion of Ki67+ cell progeny is blocked by long-lived parietal cells (green) acting as physical barriers, resulting in slow lateral expansion (right diagram). (C) Experimental timeline and representative images of whole-mount tissue from *Rosa26-CreERT2;R26R-Confetti* mouse corpus with (right) and without (left) administration of DMP-777. The white dotted lines indicate partially labeled glands. The relative number of partially labeled glands was reduced in the DMP-777-treated sample. Red, tdimer2; green, Ki67. Scale bars: 50 μm. (D) The percentage of partially labeled glands in untreated (N = 132 glands) and DMP-777-treated (N = 45 glands) samples quantified at 3 months post-induction. The glands were pooled from 2 or 3 mice per condition. See Video S2 and Figure S3.

Despite the apparent viability of the neutral drift model, such a slow rate of IsthSC loss-and-replacement seemed incompatible with both the rapid vertical clonal expansion (Figure 1F) and the rapid early lateral expansion of some clones around the gland circumference (Figure 2C). Indeed, the invariance of the average labeled cell fraction over the long term (Figure 2F) suggested that the initial rapidly expanding clones are likely to be precursors of “long-term” clones at later time points. Therefore, to resolve the paradoxical anisotropy in the dynamics of clonal expansion, we searched for constraints in the cellular organization that might present barriers to lateral clonal expansion. High-resolution 3D reconstruction of the isthmus compartment (Figure 3A) revealed an ordered spatial organization of parietal cells (HKATPase, in green) and cycling progenitors (Ki67, in red) within the isthmus region. Small Ki67+ progenitors were located between columns of larger, non-dividing parietal cells that tightly share their plasma membranes along the length of the gland to form a structure that is approximately vertically aligned—an apparent “wall” of parietal cells (Video S2). The intercalation of Ki67+ cells between walls of long-lived parietal cells suggested that the latter may act as physical barriers against the lateral expansion of stem-cell-derived clones in the isthmus region (Figure 3B).

Video S2. Spatial Organization of Ki67+ Cells and Parietal Cells in the Isthmus Region, Related to Figure 3 This movie shows that Ki67+ proliferating cells (in red) in the isthmus region of the mouse stomach corpus glands are surrounded by large, long-lived parietal cells (in green).

To assess this possibility, we eliminated parietal cells by DMP-777 treatment (Goldenring et al., 2000, Nam et al., 2010, Nomura et al., 2005) while performing *Rosa26-CreERT2;R26R-Confetti* labeling and lineage tracing. One oral administration of DMP-777 (7 mg/20 g body weight) was sufficient to induce acute loss of parietal cells, which were almost completely restored within 2 weeks (Figures S3B and S3C). This acute tissue damage resulted in no major change to the isthmus versus base lineage tracing pattern of clonal compartmentalization (Figure S3D). By contrast, lateral clonal expansion was found to be faster at 3 months post-parietal cell depletion, resulting in fewer partially labeled glands (Figure 3C): in DMP-777-treated mice, almost half of all clones (47%) reached monoclonality in the isthmus region by 3 months post-induction, compared to only 23% at the same time point in the control (Figure 3D). Indeed, it took more than 6 months for control samples to contain more than 50% of glands with monoclonal labeling in the isthmus region (Figure 2D). Together, these results indicate that the transient loss of parietal cell barriers allows IsthSCs to achieve a faster lateral drift around the gland circumference.

**Figure 4:**
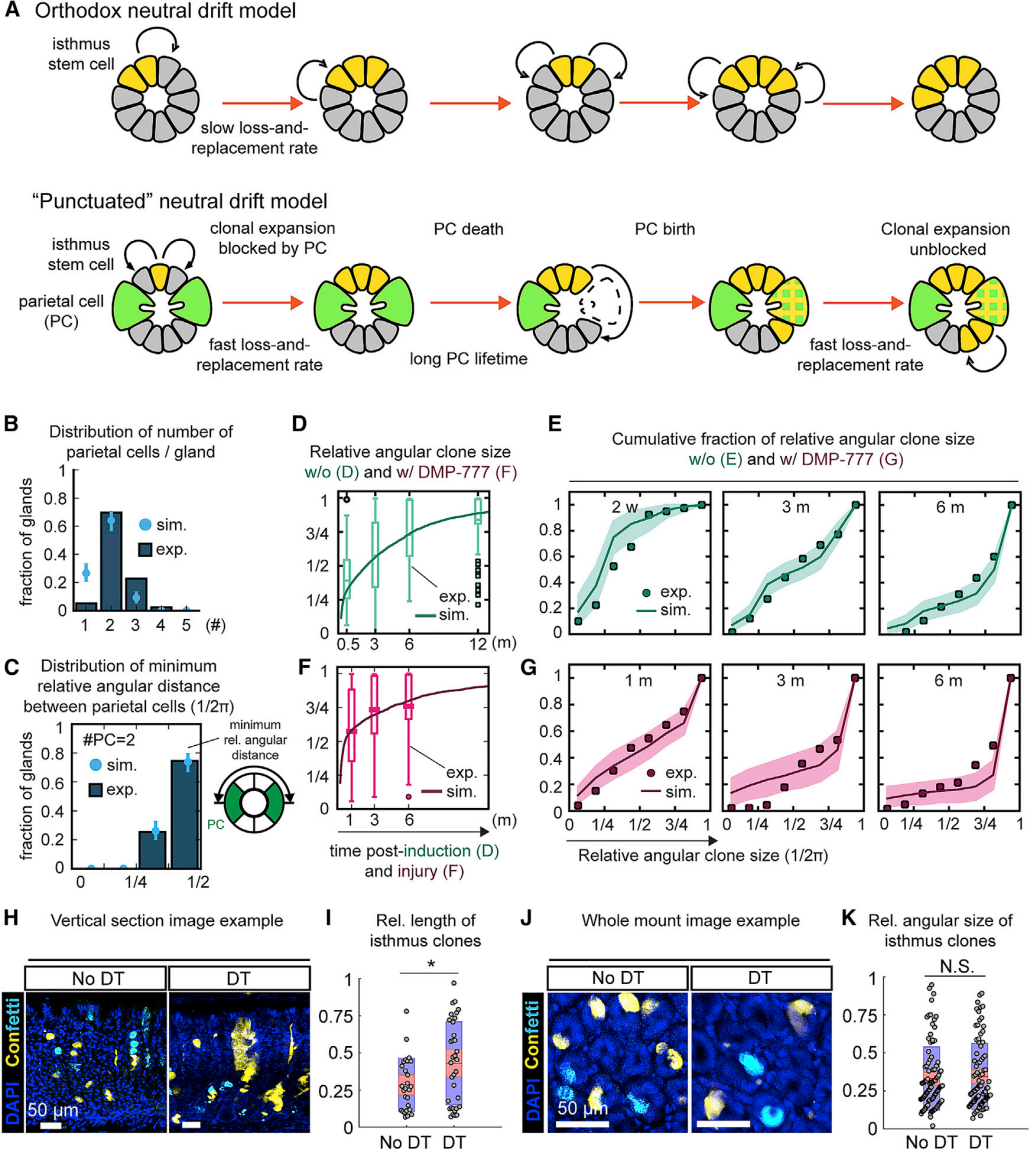
Parietal Cells Establish a Barrier Function Inhibiting the Lateral Expansion of Isthmus Clones (A) Schematic of two possible models: orthodox neutral drift model (top panel) and the “punctuated” neutral drift (PND) model (bottom panel). In the orthodox model, loss and replacement of neighboring stem cells leads to a slow drift of clones around the gland circumference. In the PND model, stem cell loss and replacement occurs rapidly in the region between parietal cells (PCs), leading to an expanded clone that persists in the region between the parietal cells. However, the loss and replacement of stem cells between neighboring regions is blocked by long-lived parietal cells. If the barrier is removed through the death of a parietal cell, clonal expansion can occur again, leading to a burst of expansion in the manner of a punctuated drift. (B) Distribution of PCs per gland section in the isthmus region. Sky blue dots and lines indicate means and SD, respectively, derived from model simulations, and dark blue bars indicate experimental data in (B) and (C). N = 175 glands. (C) Distribution of minimum relative angular distance between PCs for a value of 2 PCs per gland isthmus region. The minimum angular distance is the smallest angle between a pair of PCs. N = 122 glands. (D–G) Relative angular clone size for control (D) and DMP-777-treated (F) samples at indicated time points. Boxplots with medians in the centers represent experimental data for each condition. Solid lines represent the fit of simulated results using the PND model with and without incorporation of PCs as physical barriers, with the same model parameters but different initial conditions. Cumulative fraction of the relative angular clone size at indicated time points for control (E) and DMP-777-treated samples (G). Dots indicate the frequency as determined from experimental data. Lines indicate the fit of simulated results from the PND model with and without incorporation of PCs as physical barriers, with the same model parameters but different initial conditions. Shading displays the 95% CI around the fit of simulated data. The number of clones used for control is the same as in Figure 2. N = 99 clones (1 month), 45 clones (3 months), and 61 clones (6 months), pooled from 2 or 3 mice per time point in DMP-777-treated condition. (H and J) Representative images of vertical sections (H) and whole mounts (J) of mouse stomach corpus glands in the *Mu6-DTR;Rosa26-CreERT2;R26R-Confetti* line with or without DT at 1 month post-labeling. Cyan: mCerulean. Scale bars: 50 μm. (I and K) The relative length (I) and angular size (K) of the isthmus-anchored clones in the same samples as (H) and (J). Red line, mean; red-shaded box, 95% CI; blue-shaded box, SD. ^∗^p < 0.05. N.S.: statistically not significant (p > 0.1). Two-sample Kolmogorov-Smirnov test. N = 27 clones (no DT) and 34 clones (DT), pooled from 2 mice per condition, were analyzed in (I), and N = 98 clones (no DT) and 88 clones (DT), pooled from 2 mice per condition in (K). Note that, in the vertical sections, the red clones were not counted to avoid confusion with the dsRed signal from the *Muc6-IRES-DTR-T2A-dsRed* allele. See Figures S3, S4, and S5; Table S1; and Methods S1.

Based on this observation, we developed a minimal refinement of the neutral drift model, where rapid lateral stem cell loss and replacement is obstructed by long-lived barriers (parietal cells), which are, in turn, sporadically lost and replaced. In this model of “punctuated” neutral drift, transient parietal cell loss opens a window for lateral expansion (Figure 4A; see Methods S1), allowing isthmus clones to expand in “bursts” around the circumference of the gland. To constrain the model, we used cross-sectional images of Confetti-labeled glands (Figure S3F) to quantify the number of parietal cells (Figure 4B) and their angular distances within the isthmus (Figures 4C and S3G), finding that each cross-section hosted 2 or 3 parietal cells. Strikingly, parietal cells appeared not to be randomly positioned but were preferentially located at maximum angular distances from each other around the circumference of the gland (Figures 4C and S3G). We incorporated this observation into our model by demanding that, when born sporadically, barrier segments (viz. parietal cells) would tend to maximize the distance between each other. Based on the inferred dynamics, we questioned whether the model could predict the number distribution and relative positions of parietal cells. By tuning the relative rates of parietal cell loss and replacement, we found that we could constrain the predicted distributions of the numbers (Figure 4B) and relative positions (Figures 4C and S3G) of parietal cells to those observed experimentally (Methods S1).

We then challenged whether the difference in the dynamics of lateral expansion between the control and DMP-777 model could be due solely to the presence or absence of parietal cells in the early phase of lateral clonal expansion. Because DMP-777 treatment temporarily removes parietal cells, we reasoned that the recovery phase would provide an extended window in which isthmus clones could expand laterally. The remaining model parameters were determined from average properties of clones and glands in both control and DMP-777 experiments, which in the model are represented by different initial conditions for the presence or absence of barrier segments (Methods S1). Using only 7 data points (viz. average clone sizes in Figures 4D, 4F, and S3I) to determine the parameters, we could fit the whole range of clone behaviors, from the dynamics of average clone expansion and loss to the size distribution and accumulation of monoclonal glands (Figures 4E, 4G, S3J, and S3K). In particular, the model of punctuated neutral drift provided an intuitive explanation for the changing speed of effective clonal expansion, with rapid early growth within parietal barriers leading to the slower growth phase across boundaries (Figures 1F and 4D). Within this paradigm, the loss-and-replacement rate of isthmus cells, $\lambda /N^{2}=0.3/\text{month}$, was some ten-fold faster than that inferred from the orthodox neutral drift model, and the inferred lifetime for barrier segments in the range of a few months was compatible with the expected lifetime of parietal cells (for which an average lifespan of about 54 days has been reported in mice; Karam, 2010). Importantly, this model resolves the paradoxical anisotropy of clone expansion and, in common with ISCs, predicts that the loss-and-replacement rate of IsthSCs is comparable to their cell cycle rate.

To further challenge the model, and rule out the possibility that accelerated lateral expansion under DMP-777 treatment may be due to the effect of injury on IsthSC proliferation, we considered the effect of a perturbation that could discriminate between vertical and lateral clonal expansion. In the orthodox model, the major determinant of lateral clone expansion is the stem cell loss-and-replacement rate, a property of the stem cell compartment. As depicted in Figures S4A–S4D, if the loss-and-replacement rate increases, the lateral clone expansion is accelerated (Figures S4C and S4D), leading to faster clone fixation of the gland as compared to the control (Figures S4B and S4D). However, in the punctuated drift model (Figures S4E–S4J), an increased loss-and-replacement rate results in more or less the same rate of angular clonal expansion (Figures S4G and S4H) as compared to the control (Figures S4F and S4H), while enhancing the speed of vertical clone expansion (Figure S4G). By contrast, a decreased parietal cell lifespan (or indeed transient parietal cell loss by DMP-777) can cause accelerated lateral clone expansion (Figures S4I and S4J) as compared to the control (Figures S4F and S4J), although not affecting the vertical expansion dynamics (Figure S4I), a behavior consistent with our data (Figure S3D). It therefore follows that the dynamics of IsthSCs in the punctuated model is determined by the physical properties of the niche, not by intrinsic competition within the stem cell compartment alone.

To validate the different predictions of the two models, we made use of the *Muc6-DTR;Rosa26-CreERT2;R26R-Confetti* system (Figure S5A). Because Muc6 is a marker for neck cells (positioned close to IsthSCs), we could specifically deplete neck cells upon diphtheria toxin (DT) administration while preserving parietal cells (Figures S5B–S5D), which results in an injury response of IsthSCs as well as BSCs (Figure S5E). In this case, if the injury itself were the main reason that the lateral expansion is accelerated, rather than the loss of parietal cells, we should also observe increased lateral expansion of the isthmus-anchored clones in *Muc6-DTR;Rosa26-CreERT2;R26R-Confetti* mice treated with DT, even in the presence of the parietal cell barrier. Therefore, we treated mice with DT at 1 week post-induction by TAM. The degree of neck cell depletion by DT (Figures S5F and S5G) was significant and, by degree, comparable to that of parietal cell loss by DMP-777 treatment (Figures S3B and S3C). Mice were examined at 1 month post-TAM induction (viz. 3 weeks post-DT treatment). We found that the vertical expansion of isthmus clones was significantly increased with DT treatment compared to controls (Figures 4H and 4I), and the lateral expansion of isthmus clones was almost the same (Figures 4J and 4K), consistent with the punctuated drift model (Figures S4G, S4H, and S5H). These findings confirm that it is the removal of parietal cell barriers that results in accelerated lateral expansion of clones in the DMP-777 experiment.

### Rapidly Cycling Isthmus Progenitors Can Maintain Long-Term Self-Renewal Potential

**Figure 5:**
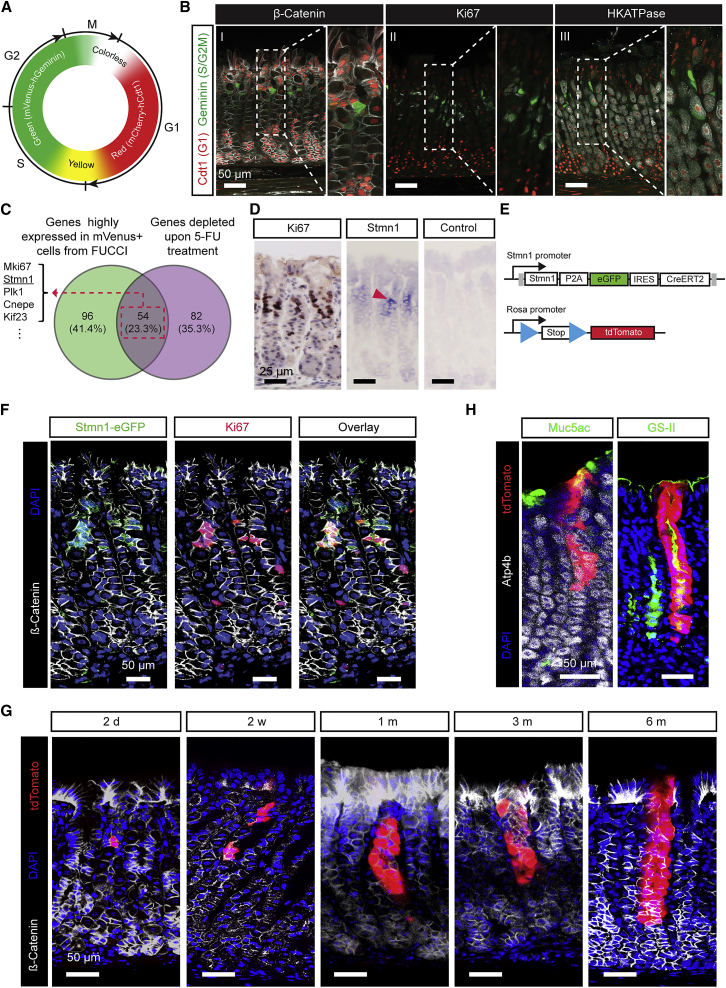
Rapidly Cycling Isthmus Progenitors Can Maintain Long-Term Self-Renewal Potential (A) Schematic showing the fluorescence signal associated with each phase of the cell cycle in the *Rosa26-Fucci2a* mouse. (B) Representative confocal images of stomach corpus gland sections (150 μm) of *Rosa26-Fucci2a* mice. Red, hCdt1-mCherry(30/120-G1); green, hGem(1/110-S/G2/M); grey, β-catenin (I), Ki67 (II), HKATPase (III). Scale bars: 50 μm. (C) Venn diagram showing the overlap between genes more highly expressed in isolated proliferative isthmus cells and genes downregulated following 5-FU treatment, taken to be prospective candidate markers for proliferating isthmus cells. (D) Immunohistochemical staining for Ki67, counterstained with Mayer’s hematoxylin (leftmost panel). *In situ* hybridization with *Stmn1* anti-sense (middle panel) and *Stmn1* sense negative control (rightmost panel) probes in mouse stomach corpus sections are shown. *Stmn1* mRNA was restricted to the isthmus region delineated by Ki67 staining. Scale bars: 25 μm. (E) Schematic of the genetic strategy to trace Stmn1+ cell-derived progeny in the corpus gland. (F) Representative confocal images of the stomach corpus of *Stmn1-CreERT2* mice. Red, Ki67; green, EGFP. Scale bars: 50 μm. (G) Representative confocal images of 150-μm-thick sections of the stomach corpus of *Stmn1-CreERT2;R26R-tdTomato* mice at 2 days, 2 weeks, 1 month, 3 months, and 6 months post-injection of 0.5–1 mg tamoxifen/20 g mouse body weight. Labeling of clones in the isthmus region with tdTomato can be detected as early as 2 days post-injection. Red, tdTomato. Scale bars: 50 μm. (H) Representative confocal images of the mouse stomach corpus glands of *Stmn1-CreERT2;R26R-tdTomato* mice at 5 months post-labeling. Red, tdTomato for labeled cells; grey, Atp4b for parietal cells; green, Muc5ac for pit cells and GS-II for neck cells. Scale bars: 50 μm. See Figure S6 and Tables S2 and S3.

Based on the proliferative activity of IsthSCs, we then searched for reliable markers to characterize actively cycling cells in the gastric isthmus. To this end, we employed the fluorescent ubiquitination-based cell cycle indicator (Fucci) ubiquitous mouse model (*Rosa26-Fucci2a*) to isolate proliferating isthmus cells for expression analysis (Mort et al., 2014). This mouse model utilizes the reciprocal degradation during the cell cycle of truncated forms of Cdt1 (hCdt1(30/1/20)) and Geminin (hGem(1/110)) fused to the fluorescent proteins Venus (green) and Cherry (red), respectively (Figure 5A). The green signal (mVenus-hGem(1/110)) marks cells in S-G2-M phase. In the stomach corpus epithelium, the Fucci mouse indicated a prominent band of proliferating progenitors marked by green signal in the isthmus region (Figure 5B). Using fluorescence-activated cell sorting (FACS), we isolated S-G2-M phase cells (R3, Venus+) from the epithelial cell fraction (R2, E-Cadherin+) (Figure S6A) and performed bulk RNA-seq analysis. By integrating this bulk RNA-seq data with additional criteria, we identified two marker candidates for the actively cycling isthmus progenitor population (Figures 5C and S6B; see STAR Methods). One candidate was the widely used proliferation marker, Mki67 (Ki67), and the other was Stathmin1 (Stmn1), the expression of which were confirmed by immunohistochemistry and *in situ* hybridization, respectively (Figure 5D).

To validate our hypothesis that the proliferating isthmus progenitor population includes IsthSCs, we generated a *Stmn1-P2A-eGFP-IRES-CreERT2* mouse line (Figure 5E). EGFP expression of *Stmn1-P2A-eGFP-IRES-CreERT2* (*Stmn1-iCre*) mice was highly specific to Ki67+-proliferating isthmus progenitors (Figure 5F). We checked that there was no induction in *Stmn1-iCre* mice without TAM treatment (Figure S6C). Upon TAM administration, clones induced by the *Stmn1-iCre* model began to appear in proximity to the isthmus region with rapid migration and (bidirectional) expansion of clones (Figures 5G and S6D). Only a small fraction of clones was observed in the base region (Figure S6D), most likely arising from the rare proliferative activity of slowly cycling chief cells at the time of induction. At subsequent time points (0.5–6 months), *Stmn1-iCre*-labeled clones exhibited long-term lineage tracing in the upper part of corpus glands, generating pit, neck, and parietal cells (Figures 5G and 5H). *Mki67*^*tm2.1(cre/ERT2)Cle*^*;R26R-RFP* (*Ki67-iCre*)-mediated tracing experiments also yielded similar long-term, pit-isthmus-neck-spanning clones up to 6 months post-induction (Figure S6E). The isthmus clones in *Ki67-iCre* lines also showed slow lateral expansion, as observed from the ubiquitous promoter, and DMP-777-mediated depletion of parietal cells resulted in accelerated drift (Figures S6F and S6G). Together, these results provide further evidence that actively cycling cells in the isthmus contain IsthSCs.

### scRNA-Seq Analysis Defines the Molecular Signature and Lineage Relationships of IsthSCs

**Figure 6:**
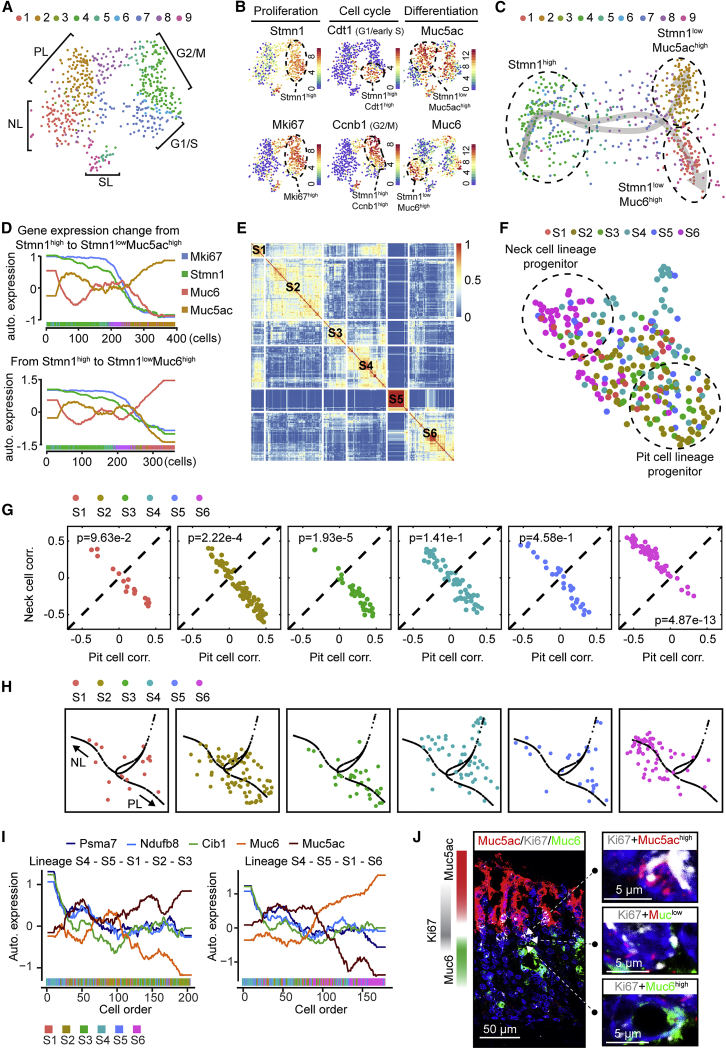
scRNA-Seq Analysis Defines the Molecular Signature and Lineage Relationships of Actively Cycling IsthSCs (A) t-SNE map showing the result of the consensus clustering (Kiselev et al., 2017) applied to scRNA-seq data from Stmn1+ cells. Based on marker gene expression, 9 clusters were classified into 5 states or lineages: G1/S phase; G2/M phase; pit cell lineage (PL); neck cell lineage (NL); and secretory cell lineage (SL). (B) t-SNE maps of Stmn1+ cells showing expression of representative genes for cell proliferation (Stmn1 and Mki67), cell cycle (Cdt1 and Ccnb1), and differentiation (Muc5ac and Muc6). The color bar indicates log2-transformed normalized read counts. (C) Pseudotime trajectory of Stmn1+ cells inferred by Slingshot (Street et al., 2018). This algorithm suggests that Stmn1^high^ cells differentiate toward two sublineages, pit (Stmn1^low^ Muc5ac^high^) and neck (Stmn1^low^ Muc6^high^) cells. (D) Expression of marker genes along the pseudotime trajectory in (C). Proliferation marker (Mki67 and Stmn1) expression decreases along the trajectory from Stmn1^high^ subpopulations toward Stmn1^low^ Muc5ac^high^ and Stmn1^low^ Muc6^high^ subpopulations. Muc5ac expression increases and Muc6 expression decreases along the path toward pit cell identity (top panel) and vice versa toward neck cell identity (bottom panel), reflecting bipotential differentiation of progeny derived from IsthSCs. Gene expression is represented as auto-scaled, log2-transformed normalized read counts. (E) Consensus matrix from SC3 clustering that justifies the identification of 6 subclusters (S1–S6) from the Stmn1^high^ cluster (top left in B). Similarity 0 (blue) in the color bar means that the two cells are always assigned to distinct clusters, whereas similarity 1 (red) means that the two cells are always assigned to the same cluster. (F) UMAP map showing the 6 subclusters (S1–S6) of the Stmn1^high^ cluster. Based on marker gene expression, S2 and S3 are primed toward the PL, whereas S6 are primed toward the NL. (G) Scatterplots of the correlation with the representative pit cell (pit cell correlation) and neck cell (neck cell correlation) for the 6 subclusters S1–S6. The p values (p) from the binomial test indicate the statistical significance of the bias of the correlations of a cluster between pit cell and neck cell lineages. N = 18 cells (S1), 82 cells (S2), 32 cells (S3), 56 cells (S4), 29 cells (S5), and 65 cells (S6) were used for the binomial test. (H) The subclusters S1–S6 in the pseudotime trajectory inferred by Slingshot where undifferentiated IsthSCs are primed toward early sublineage-restricted progenitors. (I) Gene expression trajectories along the pseudotime trajectory for two lineages. The lineages S4-S5-S1-S2-S3 and S4-S5-S1-S6 are pit cell and neck cell lineages, respectively. The represented expression values are log2-transformed normalized read counts followed by the z transform. The subcluster ID of cells is denoted by colors along the pseudotime orders in the bar on the x axis. Muc5ac and Muc6 show opposite behaviors in the two lineages. The expression of Cib1, Ndufb8, and Psma7 is highest at the start of pseudotime and decreases along the trajectory leading to early sublineage restriction. (J) Examples of undifferentiated IsthSCs and early sublineage-restricted progenitors in the isthmus region of the mouse stomach corpus glands based on mRNA expression detected by RNAscope. Red, *Muc5ac*; green, *Muc6*; grey, *Ki67*. “Ki67+Muc5ac^high^,” “Ki67+Muc^low^,” and “Ki67+Muc6^high^” indicate (1) Ki67+ cells expressing Muc5ac at high levels (PL progenitor); (2) Ki67+ cells expressing both Muc5ac and Muc6 at low levels, but not zero (IsthSC); and (3) Ki67+ cells expressing Muc6 at high levels (NL progenitor). See Figure S7 and Table S4.

To identify cells with IsthSC characteristics within the Stmn1+ isthmus population, we performed scRNA-seq analysis with sorted Stmn1-eGFP+ cells (Figure S6H). We identified 9 clusters using consensus-based clustering (Kiselev et al., 2017; Figure 6A). 46% of cells (clusters 4–7 in Figure 6A) were classified as belonging to the Stmn1^high^ population, and others (clusters 1–3, 8, and 9 in Figure 6A) were Stmn1^low^ (Figure 6B, top left panel). The appearance of the latter pool is likely a reflection of the stability of the EGFP protein, suggesting that Stmn1^high^ cells are precursors of Stmn1^low^ cells. Ki67 expression largely overlapped with Stmn1 (Figure 6B, bottom left panel), confirming that the Stmn1+ and Ki67+ populations are nearly identical at the transcriptional level and mark highly proliferative cells (Figure S7A).

Based on the expression of cell cycle phase-specific genes in the t-distributed stochastic neighbor embedding (t-SNE) plot, the Stmn1^high^ population could be partitioned largely into two subpopulations marked by expression of Cdt1 (G1/early S) and Ccnb1 (G2/M; Figures 6B, middle panels, and S7B–S7D). By contrast, the Stmn1^low^ population could be cleanly segregated by the expression of pit (Muc5ac) and neck (Muc6) markers (Figures 6B, right panels, S7E, and S7F), implying that, depending on their differentiation path, Stmn1^low^ cells migrate upward or downward from the isthmus region to become pit and neck cells, respectively. This became even more obvious in the pseudotime analysis (Figure 6C), showing divergent differentiation trajectories of the Stmn1^high^ population toward pit or neck cell fates (Figures 6C, 6D, S7G, and S7H; STAR Methods).

Alongside the strong cell cycle signature (Figure 6B), the Stmn1^high^ cluster showed heterogeneous but structured expression of the differentiation markers Muc5ac and Muc6, with cells belonging to the four groups: (1) Cdt1^high^ Muc5ac^high^; (2) Ccnb1^high^ Muc5ac^high^; (3) Cdt1^high^ Muc6^high^; and (4) Ccnb1^high^ Muc6^high^ (Figure S7I). We reasoned that expression of these differentiation markers within the actively cycling population may be evidence of early sublineage restriction of progenitors. Based on this observation, we regressed out the cell cycle signature (Butler et al., 2018). As a result, the Stmn1^high^ cluster segregated into 6 subclusters (S1–S6; Figures 6E and 6F), showing different degrees of bias toward pit or neck cell lineages (Figure 6G). We then measured the distance between each cell in the Stmn1^high^ cluster and neck and pit cells based on their correlation of gene expression (see STAR Methods). From this analysis, we found that subclusters S2 and S3 have higher correlation with pit cells than neck cells, with the degree and statistical significance of the bias indicated by p < 0.01 (Figure 6G). Conversely, subcluster S6 showed a higher correlation with neck cells than pit cells (Figure 6G). This suggests that these three subclusters constitute progenitor populations that have already become restricted to the respective sublineages. Subclusters S1, S4, and S5 had relatively unbiased signatures with respect to neck and pit cell lineages, implying that these might constitute the undifferentiated stem cell compartment. However, even with the overall pattern of bias toward pit or neck cell sublineages, cells in subclusters S1–S6 were almost evenly spread along the diagonal line of the pit-neck cell correlation spaces, indicative of transcriptional heterogeneity within the individual subclusters (Figure 6G).

To confirm the correlation analysis and refine the distinction between the subclusters, we performed pseudotime analysis for the 6 subclusters (STAR Methods). Accordingly, we inferred two distinct lineages: (1) S4 → S5 → S1 → S2 → S3 leading to the pit cell lineage and (2) S4 → S5 → S1 → S6 leading to the neck cell lineage (Figure 6H). Next, we examined changes in gene expression along the pseudotime trajectory to define molecular signatures of the putative IsthSC and progenitor compartments. Distinct expression patterns were found that peak in early or late phases of the pseudotime trajectory (Figure 6I; Table S4). Differentiation-associated marker gene expression (viz. Muc5ac and Muc6) indicated that cells in the pseudotime trajectory progress toward differentiation. Some genes showed enriched expression in the putative IsthSC clusters (S4 and S5), including Cib1, Ndufb8, and Psma7 (Figure 6I), though their expression patterns were not exclusive to the isthmus compartment. Moreover, the activation of canonical stem cell signaling pathways, such as Wnt or Notch, were not observed in the IsthSC clusters (S4 and S5).

To corroborate the results of the scRNA-seq analysis, we examined the spatial organization of cells based on the expression patterns of defined clusters. To this end, we used RNAscope (Wang et al., 2012) to determine the expression pattern of Muc5ac, Muc6, and Ki67. As predicted from the single-cell data, Ki67+ cells in the isthmus region showed robust expression of Muc5ac or Muc6, consistent with sublineage-restricted progenitors. Notably, between these domains, we found Ki67+ cells showing low levels of co-expression of both Muc5ac and Muc6, consistent with the uncommitted nature of the putative IsthSCs identified by scRNA-seq (Figure 6J). These results indicate that cells in subclusters S4 and S5 are located in the isthmus center, consistent with a model in which centrally located IsthSCs support bidirectional output of differentiating progeny.

Lastly, we analyzed well-known gastric markers in the scRNA-seq data of Pgc+ gastric corpus chief cells sorted from *Pgc-IRES-DTR-T2A-dsRed* knockin mice (Figures S7J and S7K). Although it is primarily known as a chief cell marker, Pgc was found to be broadly expressed in many different gastric cell types except parietal cells. Based on cell-type-specific markers, the t-SNE plot recapitulated the anatomical localization of the stomach corpus gland with chief cells at the base, neck cells in the lower middle part, and pit cells at the top of the gland (Figure S7L). Both Ki67+ and Stmn1+ populations form a highly specific group of cells, and Lgr5+ cells are within the Gif+ chief cell domain, as recently reported (Leushacke et al., 2017; Figure S7M). However, other markers, such as Sox2, Runx1, Lrig1, Mist1 (Bhlha15), and Bmi1, showed a very broad pattern of expression in our single-cell data (Figure S7M).

## Discussion

**Figure 7:**
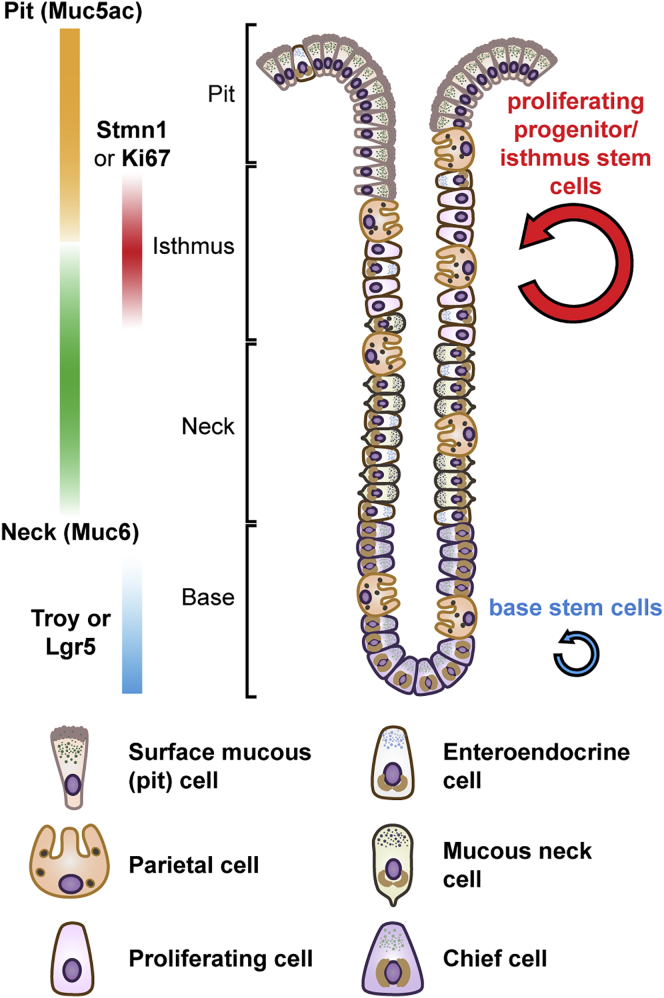
The Gastric Corpus Is Maintained by Two Stem Cell Populations Schematic of the mouse gastric corpus gland. The gastric corpus is compartmentalized into base and isthmus regions maintained by two discrete stem cell populations. IsthSCs are multipotent and actively cycling, maintaining the pit-isthmus-neck regions through a process of stochastic self-renewal. IsthSCs reside in a narrow zone between the pit and neck regions and are characterized by co-expression at low levels of the differentiation markers Muc5ac and Muc6, as well as high expression of the cell cycle markers Stmn1 and Ki67. As IsthSCs become displaced upward or downward from the stem cell zone, they become sublineage restricted, upregulating expression of Muc5ac or Muc6, before terminal differentiation into the respective cell types. The gland base is maintained by Troy+ or Lgr5+ (chief) stem cells, which are mostly quiescent during homeostasis and persist long-term. See Figure S7.

Based on the analysis of clonal data (in normal and perturbed states) and single-cell profiling, we propose a unified framework in which the epithelium of gastric corpus glands are maintained by two distinct stem cell populations: an actively proliferating stem cell population in the isthmus region with a broad expression signature and a quiescent reserve stem cell population in the base, marked by expression of Troy or Lgr5 (Figure 7). Although the existence of a quiescent IsthSC population cannot be ruled out, quantitative analysis of clonal fate data demonstrates that actively cycling, multipotent IsthSCs are capable of long-term self-renewal.

Through a process of “punctuated” neutral drift, our results show that the loss and replacement of IsthSCs lead to rapid vertical clone expansion, and the lateral expansion of clones is impeded, as previously observed (Nomura et al., 1998), by the physical barrier of long-lived parietal cells. Only when a parietal cell is lost can clones expand laterally around the gland circumference, leading eventually to clonal fixation of the isthmus region. Indeed, this behavior echoes that reported in human colon (Stamp et al., 2018, Nicholson et al., 2018), suggesting that a similar barrier-inhibited—or punctuated—drift mechanism may be operative in other glandular contexts. Although the function of barrier restriction is unclear, the geometric arrangement of parietal cells may prevent them from being “pushed out” into the lumen by the tide of migrating Muc5ac+ differentiating cells during the course of homeostatic turnover.

To address the molecular identity of corpus stem cells during homeostasis, we generated scRNA-seq data from Stmn1-eGFP+ cells containing both proliferating IsthSCs and their early differentiating progeny. We found evidence for substructure within the Stmn1^high^ cluster, which allowed us to abstract a restricted stem cell signature as well as relationships between IsthSCs and their immediate sublineage-restricted progenitor cell progeny. Our findings do not reveal an exclusive and definitive stem cell marker: rather, we find genes that are enriched in, but not exclusive to, IsthSCs. These results suggest that, as in the intestinal crypt (Ritsma et al., 2014), the stem cell signature may be broad, with varying degrees of renewal potential linked to exposure to localized niche factors associated with neighboring epithelial and/or stromal cell types. As IsthSCs divide, others may become displaced from the niche region, moving down into the neck region or up toward the pit region, becoming sublineage restricted to the neck or pit cell lineages. Future studies must target the origin and repertoire of such niche factors that support the self-renewal activity and multipotency of the IsthSC compartment.

## STAR★Methods

### Key Resources Table

**Table:** 

REAGENT or RESOURCE	SOURCE	IDENTIFIER
**Antibodies**		

rabbit anti-Ki67	A. Menarini	Cat#MP-325-CRM1
β-Catenin (L54E2) Mouse mAb (Alexa Fluor® 647 Conjugate)	Cell Signaling Technology	Cat#4627S; RRID:AB_10691326
chicken anti-EGFP	abcam	Cat#ab13970; RRID:AB_300798
mouse anti-MUC5AC	Thermo Fisher Scientific	Cat#MA5-12178; RRID:AB_10978001
Alexa 488-conjugated GS-II	Thermo Fisher Scientific	Cat#L21415
rabbit anti-H-K-ATPase	Santa Cruz	Cat#sc-84304; RRID:AB_2061759
Alexa 555-conjugated rabbit anti-ATP4b	bioss	Cat#bs-2433R-AF555
Alexa 647-conjugated rabbit anti-ATP4b	bioss	Cat#bs-2433R-AF647
Dako EnVision+ System-HRP (DAB) For Use with Rabbit Primary Antibodies	DAKO	Cat#K4010
Donkey anti-Rabbit IgG (H+L) Secondary Antibody, Alexa Fluor 488	Thermo Fisher Scientific	Cat#A-21206; RRID:AB_2535792
Donkey Anti-Rabbit IgG (H L) Antibody, Alexa Fluor 647	Thermo Fisher Scientific	Cat#A-31573; RRID:AB_2536183
ANTI-DIGOXIGENIN AP-CONJUGATE	Roche	Cat#11093274910; RRID:AB_514497
Alexa Fluor® 647 anti-mouse/human CD324 (E-Cadherin) Antibody	BioLegend	Cat#147308; RRID:AB_2563955

**Chemicals, Peptides, and Recombinant Proteins**		

Normal Donkey Serum	Jackson ImmunoResearch	Cat#017-000-121
DMEM, high glucose, HEPES, no phenol red	Thermo Fisher Scientific	Cat#21063-029
RapiClear 1.52	CamBioScience	Cat#RC152002
DNase I (RNase-free)	Thermo Fisher Scientific	Cat#AM2222
4′,6-Diamidino-2-phenylindole dihydrochloride	Sigma-Aldrich	Cat#D8417
Certified Low Melt Agarose	BIO-RAD	Cat#1613112
Dispase II	Life Technologies	Cat#17105-041
Pancreatin from porcine pancreas	Sigman-Aldrich	Cat#P3292-25G
DMP-777	Matrix Scientific	Cat#96053
Tamoxifen	Sigma-Aldrich	Cat#T5648
Doxycycline	Sigma-Aldrich	Cat#9891
Diphtheria toxin	Sigma-Aldrich	Cat#D0564
Blocking reagent	Roche	Cat#11096176001

**Critical Commercial Assays**		

QuantSeq 3′ mRNA-Seq Library Prep Kit FWD for Illumina	Lexogen	Cat#015
TSA Fluorescein System	Perkin Elmer	Cat#NEL701A001KT
DIG RNA Labeling Mix	Roche	Cat#11277073910
RNAscope® Multiplex Fluorescent Reagent Kit v2	Advanced Cell Diagnostics	Cat#323100
TSA kits TSA Plus Cyanine 3 and Cyanine 5 System	Perkin Elmer	Cat#NEL752001KT
PicoPure® RNA Isolation Kit	Life Technologies	Cat#KIT0204

**Deposited Data**		

single-cell RNA-seq	This paper	ArrayExpress: E-MTAB-6879
bulk RNA-seq data	This paper	ArrayExpress: E-MTAB-6850

**Experimental Models: Organisms/Strains**		

Rosa26-rtTA	Jackson Laboratory	Cat#006965; RRID:IMSR_JAX:006965
*Muc6-IRES-DTR-T2A-dsRed*	This paper	N/A
*Rosa26-StopFlox-tdTomato*	Jackson Laboratory	Cat#007914; RRID:IMSR_JAX:007914
*Mki67*^*tm2.1(cre/ERT2)Cle*^	Basak et al., 2018	Cat#029803; RRID:IMSR_JAX:029803
*Stmn1-P2A-eGFP-IRES-CreERT2*	This paper	N/A
*CAGG-CreERT2*	Jackson Laboratory	Cat#004682; RRID:IMSR_JAX:004682
*R26-Fucci2a*	Mort et al., 2014	MGI ID: 6193738
*TetO-Cre*	Jackson Laboratory	Cat#011004; RRID:IMSR_JAX:011004
*UBC-CreERT2*	Jackson Laboratory	Cat#008085; RRID:IMSR_JAX:008085
*Pgc-IRES-DTR-T2A-dsRed*	This paper	N/A
*Rosa26-CreERT2*	Jackson Laboratory	Cat#008463; RRID:IMSR_JAX:008463
*Rosa26-StopFlox-Confetti*	Jackson Laboratory	Cat#013731; RRID:IMSR_JAX:013731

**Oligonucleotides**		

Probe-Mm-Muc5ac-C2	Advanced Cell Diagnostics	Cat#448471-C
Probe-Mm-Mki67-C3	Advanced Cell Diagnostics	Cat#416771-C3
Probe-Mm-Muc6	Advanced Cell Diagnostics	Cat#448481

**Software and Algorithms**		

DiffusionMap (v1.1.0)	Joseph Richards	https://rdrr.io/cran/diffusionMap/
slingshot (v1.0.0)	Street et al., 2018	https://github.com/kstreet13/slingshot
HTseq (v0.7.2)	Anders et al., 2015	https://github.com/simon-anders/htseq
monocle (v2.6.4)	Qiu et al., 2017	https://bioconductor.org/packages/release/bioc/html/monocle.html
destiny (v2.6.2)	Haghverdi et al., 2016	https://bioconductor.org/packages/release/bioc/html/destiny.html
umap (v0.2.0.0)	Becht et al., 2019	https://cran.r-project.org/web/packages/umap/index.html
amap (v0.8-16)	Antoine Lucas	https://cran.r-project.org/web/packages/amap/index.html
pheatmap (v1.0.12)	Raivo Kolde	https://cran.r-project.org/web/packages/pheatmap/index.html
Imaris 8.1.0	Bitplane	https://imaris.oxinst.com/
EdgeR (v3.26.4)	Robinson et al., 2010	https://bioconductor.org/packages/release/bioc/html/edgeR.html
DESeq	Anders and Huber, 2010	http://bioconductor.org/packages/release/bioc/html/DESeq.html
ImageJ	Schneider et al., 2012	https://imagej.net/Fiji/Downloads
scran (v1.6.9)	Lun et al., 2016	https://bioconductor.org/packages/release/bioc/html/scran.html
Rtsne (v0.13)	van der Maaten, 2014	https://github.com/jkrijthe/Rtsne
STAR (v2.5.2b)	Dobin et al., 2013	https://github.com/alexdobin/STAR
samtools (v1.4)	Li et al., 2009	https://github.com/samtools/samtools
SC3 (v1.7.7)	Kiselev et al., 2017	http://bioconductor.org/packages/release/bioc/html/SC3.html
Seurat (v2.3.0)	Butler et al., 2018	https://github.com/satijalab/seurat
Mathematica 11	Wolfram	http://www.wolfram.com/mathematica/
Python (v3.7.3)	Python Software Foundation	https://www.python.org/
gfortran (v6.3)	GNU Fortran project	https://gcc.gnu.org/wiki/GFortranBinaries
Datasets/codes for modeling and single-cell RNA-seq analysis	This paper	https://github.com/scg-dgist/CELL-STEM-CELL-isthmus-stem-cells

**Other**		

VT1000S	Leica	Cat#14047235612
iSpacer 0.5mm	CamBioScience	Cat#IS017

### Lead Contact and Materials Availability

Further information and requests for resources and reagents should be directed to, and will be fulfilled by, the Lead Contact, Dr. Bon-Kyoung Koo (bonkyoung.koo@imba.oeaw.ac.at). Mouse lines generated during this study will be freely available to academic researchers upon request.

### Experimental Model and Subject Details

#### Animals

All ubiquitous, inducible Cre lines (*Rosa26-CreERT2*, *UBC-CreERT2*, *CAGG-CreERT2*, *TetO-Cre*) and *Rosa26-rtTA*, *Rosa26-StopFlox-Confetti* and *Rosa26-StopFlox-tdTomato* lines were obtained from The Jackson Laboratory. The *Rosa26-Fucci2a* (MGI ID: 6193738) line was a generous gift from Dr. Richard Mort (Mort et al., 2014). The *Mki67*^*tm2.1(cre/ERT2)Cle*^ (stock no: 029803) line was obtained from Dr. Hans Clevers (Basak et al., 2018). *Stmn1-P2A-eGFP-IRES-CreERT2* targeting vectors were generated by gene synthesis. The *DTR-T2A-dsRed* cassette used for vector generation was a generous gift from Dr. Nobuo Sasaki (Sasaki et al., 2016). Strategy development and targeting vector generation for *IRES-DTR-T2A-dsRed* constructs was performed with the help of the WT–MRC Stem Cell Institute Advanced Vector Design and Recombineering facility. Subsequent modifications to the resulting vector were implemented by In-Fusion cloning.

#### Animal treatments

Tamoxifen (Sigma, Cat. T5648) in corn oil was injected into mice intraperitoneally in order to induce lineage tracing. The dose of tamoxifen used for different lines was determined based on recombination efficiency in the corresponding reporter lines and was as follows (mg of tamoxifen per 20 g of mouse body weight): 5 mg for *UBC-CreERT2;Rosa26-StopFlox-Confetti*; three consecutive doses of 0.75 mg for *CAGG-CreERT2;Rosa26-StopFlox-Confetti;* 3 mg for *Rosa26-CreERT2;Rosa26-StopFlox-Confetti* and *Muc6-DTR;Rosa26-CreERT2;Rosa26-StopFlox-Confetti*; 2.5 mg for *Ki67-IRES-CreERT2;Rosa26-StopFlox-RFP*; 0.5 or 1 mg for *Stmn1-P2A-eGFP-IRES-CreERT2;Rosa26-StopFlox-tdTomato*. *TetO-Cre;Rosa26-rtTA;Rosa26-StopFlox-Confetti* mice were injected intraperitoneally with 2 mg doxycycline (Sigma, Cat. 9891) in PBS per 20 g of mouse body weight. Parietal cell depletion was performed by administration by oral gavage of 7 mg DMP-777 (Matrix Scientific, Cat. 096053) per 20 g mouse body weight. 20 ng diphtheria toxin (DT; Sigma, Cat. D0564) per 20 g of mouse body weight was injected intraperitoneally to ablate the neck cells of *Muc6-DTR;Rosa26-CreERT2;Rosa26-StopFlox-Confetti* mice. Stomach samples were obtained from mice at different time-points, as indicated, following either a single or three consecutive injections of tamoxifen alone or in combination with subsequent administration of DMP-777 or DT. All mice were group housed under specific pathogen-free conditions and had not previously undergone any procedures. We used both male and female mice (8-14 weeks of age) in all our experiments: the influence of sex has not been considered in this study, as homeostatic tissue turnover exists in both sexes. All procedures were performed according to United Kingdom Home Office regulations and local animal welfare committee guidelines.

### Method Details

#### Stomach preparation

Mice were euthanized by cervical dislocation and the stomach removed by dissection. The stomach was cut longitudinally following the greater curvature from the intestine to the esophagus and subsequently spread on a piece of cardboard, using needles to hold the tissue, before fixation in freshly prepared 4% PFA at 4°C overnight (O/N) (∼18h) with shaking. After fixation the stomach tissue was washed for 3 × 30 min with PBS at 4°C with shaking.

#### Whole mount immunofluorescence (IF)

Corpus whole mounts were dissected from fixed stomach samples and transferred into 1 mL blocking and permeabilization solution consisting of 5% DMSO, 0.5% Triton X-100 and 2% Normal donkey serum (NDS) in PBS in a 1.5 mL Eppendorf tube. All subsequent incubation steps were then performed at 4°C using a rotor. Whole mounts were incubated with blocking and permeabilization solution O/N. The following day, the blocking and permeabilization solution was replaced and the whole mount incubated for 7 days with primary antibody, either rabbit anti-Ki67 (1:250; A. Menarini, MP-325-CRM1) or Alexa 647-conjugated mouse anti-β-catenin (1:200; Cell Signaling Technology, 4627S), diluted in blocking solution (1% DMSO, 0.5% Triton X-100 and 2% NDS in PBS). The sample was then washed 6 times with fresh PBS over a 24h period before incubating with anti-rabbit Alexa 488-conjugated IgG (1:1000; Thermo Fisher Scientific) secondary antibody diluted in blocking solution for 7 days. Whole mounts were washed several times with PBS and incubated with 2 μg/mL DAPI in PBS for 24 h. For optical clearing, samples were transferred into a 24-well plate containing 1 mL RapiClear 1.52 (CamBioScience). To avoid tissue rolling, samples were held flat between two pieces of plastic mesh for O/N incubation at 4°C. To allow imaging from both sides, the whole mounts were mounted in RapiClear 1.52 between two class-1 cover glasses (22 × 32 mm) using a 500 μm iSpacer (CamBioScience).

#### Near-native sectioning

The corpus was dissected and embedded in 4% low melt agarose for sectioning of 100 – 150 μm thick near-native sections using the LEICA VT 1000S Vibratome.

#### IF staining of near-native sections

Near-native sections were removed from any remaining agarose using forceps and subsequently transferred to a 12-well plate into wells containing blocking and permeabilization solution (5% DMSO, 0.5% Triton X-100 and 2% NDS in PBS) and incubated O/N (∼18 h) at 4°C with shaking. The following day, the blocking and permeabilization solution was replaced with primary antibody diluted in blocking solution (1% DMSO, 0.5% Triton X-100 and 2% NDS in PBS) and the section incubated for 72-96 h at 4°C. The following primary antibodies were used: rabbit anti-Ki67 (1:250; A. Menarini, MP-325-CRM1), Alexa 647-conjugated mouse anti-β-catenin (1:200; Cell Signaling Technology, 4627S), rabbit anti-H+/K+ ATPase β Antibody (D-18) (1:300; Santa Cruz Biotechnology, sc-84304), mouse anti-MUC5AC (1:300; Thermo Fisher Scientific, MA512178), Alexa 488-conjugeted Lectin GS-II (1:500; Thermo Fisher Scientific, L21415). Sections were subsequently washed and incubated with an appropriate secondary antibody and DAPI in blocking solution (1% DMSO, 0.5% Triton X-100 and 2% NDS in PBS) for 48 h at room temperature with shaking. After washing for 3 × 45 min with PBS, sections were carefully transferred from wells to microscope slides using a brush before mounting in RapiClear 1.52 (CamBioScience). Slides were sealed and stored at 4°C in the dark before imaging by fluorescent microscopy.

#### Confocal imaging

Confocal microscopy was performed with a Leica SP5 TCS confocal microscope with 20 x dry objective and 1-2.5 fold digital zoom using the LAS AF software. The argon laser intensity was set to 30%. The X/Y resolution was set to 1024 × 1024 pixels and the Z-step size was set to 1-2.5 μm. Laser scanning frequency was set to 400 Hz and the line average was set to 2 or 3. Section images were processed and analyzed using ImageJ. Whole mount images were processed using AutoQuant X 3D deconvolution for subsequent analysis using Imaris software. Signal intensity was adjusted for analysis.

#### Immunohistochemistry in paraffin sections

Immunohistochemistry was performed according to standard protocols. Briefly, tissues were fixed in 4% PFA overnight at 4°C before paraffin embedding. Paraffin-embedded sections (5 μm) were rehydrated, and the epitopes were exposed using Tris/EDTA buffer. Sections were incubated in blocking solution (2% donkey or goat serum, 5% DMSO and 0.5% Triton X-100 in PBS) at room temperature for 2 h. The following primary antibodies were used: chicken anti-EGFP (1:500; Abcam, ab13970), rabbit anti-Ki67 (1:250; A. Menarini, MP-325-CRM1), Alexa 647-conjugated mouse anti-β-catenin (1:200; Cell Signaling Technology, 4627S), rabbit anti-H+/K+ ATPase β Antibody (D-18) (1:500; Santa Cruz, sc-84304) and Alexa 555/647-conjugated rabbit anti-ATP4b (1:200; Bioss, bs-2433R-AF555, bs-2433R-AF647). The peroxidase-conjugated secondary antibodies used were rabbit EnVisionC (DAKO) for 3,3′-diaminobenzidine HRP immunohistochemistry or anti-chicken/rabbit Alexa 488/647-conjugated IgG (1:1000; Invitrogen) for immunofluorescence.

#### *In situ* hybridization in paraffin sections

For *in situ* hybridization, both sense and antisense probes for *Stmn1* mRNA were generated by *in vitro* transcription using DIG RNA labeling mix (Roche, Cat. 11277073910) according to the manufacturer’s instructions. Stomach corpus was fixed overnight in 10% formalin, dehydrated and embedded in paraffin. Rehydrated sections (8-12 μm) were treated with 0.2 M HCl and digested in proteinase K solution. Sections were then post-fixed, treated with acetic anhydride solution and hybridized with the indicated probes for 24-72 h at 68°C in hybridization buffer consisting of 5 x SSC (pH 4.5), 50% formamide, 50 g/mL yeast transfer RNA, 0.05% CHAPS, 2% blocking powder (Roche, Cat. 11096176001), 5 mM EDTA and 50 μg/mL heparin in DEPC treated water. Sections were washed for 3 × 20 min at 65°C in 2 x SSC with 50% formamide. After another wash in Tris-buffered saline containing 0.05% Tween (TBST), sections were blocked for 30 min in TBST containing 0.5% blocking powder. Sections were subsequently incubated overnight at 4°C with alkaline phosphatase-conjugated anti-digoxigenin antibody (1:2000; Roche, Cat. 11093274910) in blocking solution. After rinsing several times using TBST, the coloring reaction was performed with BM purple.

RNAscope was performed on paraffin-embedded sections according to the manufacturer’s protocol (RNAscope® Multiplex Fluorescent Reagent Kit v2, ACDBio, 323100). The TSA kits TSA Plus Cyanine 3 and Cyanine 5 System (Perkin Elmer, NEL752001KT) and TSA Fluorescein System (Perkin Elmer, NEL701A001KT) were used for visualization. The following probes were used: Mm-Muc6, Mm-Muc5ac-C2 and Mm-Mki67-C3 (1:1000, ACDBio).

#### Cell sorting

Stomach cell sorting was performed by the WT–MRC Stem Cell Institute Flow Cytometry Facility and the WT Sanger Institute Flow Cytometry Facility for bulk RNA-seq and single-cell RNA-seq data generation, respectively. Stomachs were prepared by carefully separating the corpus from the forestomach and pylorus. Corpus tissue was divided into 4 pieces of similar size and incubated in 4 mL dissociation solution (45 U/mL Dispase II, Thermo Fisher Scientific; 0.6 mg/mL Pancreatin, Sigma; 1x Penicillin/Streptomycin in DMEM high glucose, HEPES, no phenol red, Thermo Fisher Scientific) at 37°C with shaking at 270 rpm. After dissociation the solution becomes cloudy and corpus fragments appear more transparent. All subsequent steps were then performed on ice. To avoid cell loss due to cell adhesion to pipette or tube walls, all pipettes and tubes used for pipetting the cell suspension were pre-incubated with either DMEM or PBS containing FBS. To further disrupt the tissue, the cell suspension, including corpus pieces, was pipetted up and down several times using a 10 mL pipette. The cell suspension without remaining corpus pieces was then transferred to a 15 mL Falcon tube. The corpus pieces and the dissociation tube were then washed twice with 5 mL DMEM + 10% FBS and the wash solutions combined with the cell suspension for inactivation of the dissociation reaction. Cells were centrifuged at 300 g for 5 min and resuspended in 1% FBS in PBS. The cell suspension was filtered through a 100 μm cell strainer into a pre-coated 15 mL tube. The tube and filters were washed twice with 1 mL of 1% FBS in PBS and the cell suspension was then centrifuged a second time at 300 g for 5 min. The supernatant was removed and the cell pellet was resuspended in 100 μL of antibody mix (1% FBS; 10 U/mL DNase, Promega; 1:125 Alexa Fluor® 647-conjugated anti-mouse/human CD324 (E-Cadherin) antibody, BioLegend) and incubated for 1 h on ice. Cells were washed with 3 mL of 1% FBS in PBS and filtered once more if clumps could be observed. After centrifugation at 300 g for 5 min the cell pellet was resuspended in 1 mL of 1% FBS and 10 U/mL DNase in PBS for sorting. MOFLO and BD INFLUX systems were used for sorting in the WT–MRC Stem Cell Institute Flow Cytometry Facility and the WT Sanger Institute Flow Cytometry Facility, respectively.

#### RNA purification for bulk RNA seq

For RNA purification, cells were sorted directly into 300 μL lysis buffer (Arcturus Pico Pure RNA Isolation Kit). Cell lysate was snap-frozen on dry ice and subsequently stored at −80°C. RNA purification was performed using the Arcturus Pico Pure RNA Isolation Kit (Life Technologies) following the manufacturer’s instructions.

#### Library preparation and bulk RNA sequencing

For single-end mRNA sequencing, RNA samples were extracted, and the integrity of the total RNA was confirmed using an Agilent BioAnalyzer. cDNA libraries were generated from total RNA according to QuantSeq 3′ mRNA-Seq FWD library preparation protocols. The library templates were amplified in PCR cycles, and concurrently barcoded. Purified PCR products were analyzed on a BioAnalyzer using a DNA HS chip. The lane-mixes were prepared for all samples and subjected to single-end 100 nt sequencing using an Illumina HiSeq 2500 system.

#### Generation of single-cell RNA-seq data

Library preparation was performed by the Single-Cell Genomics Core Facility of the WT Sanger Institute. Briefly, mRNA isolated from single cells was amplified using the SMARTSeq2 protocol (Picelli et al., 2014). Multiplexed sequencing libraries were prepared from amplified cDNA using Nextera XT (Illumina) and sequenced on a HiSeq 2500 running in rapid mode.

### Quantification and Statistical Analysis

#### Whole mount image scoring

Mouse corpus whole mounts were prepared for confocal microscopy as described above. Clonal expansion was analyzed using Imaris (Bitplane) software using a 3-point pie-chart contribution scoring strategy in which the first point denotes the beginning of a clone, the second point denotes the center of the corresponding gland and the third point denotes the end of the clone when following the circumference of the gland clockwise. Pie-chart clone contributions were measured in the pit region, the isthmus (high, middle, low; labeled with Ki67+ cells) and the lowest clone position. Additionally, the bottom of the gland was annotated with one measurement point. The resulting matrix was exported and clone contribution values were extracted using Excel and Mathematica. The statistical analysis of the clonal data was performed using Fortran and Python.

#### Gland section scoring

150 μm-thick sections were prepared and imaged as described above. Gland sections were scored using ImageJ. In each z stack, one reference z-plane was chosen at approximately the 30 μm mark. Glands that were present within the chosen reference z stack were subsequently analyzed for clonal contribution in the remaining z stack images. Clone length and position was measured for all clones containing 3 or more cells. All of the statistical details of experiments generating the clonal data for both vertical and lateral expansion can be found in relevant figures and their legends, including statistical tests, exact value and meaning of N, and definition of all attributes in graphs (e.g., mean, median, SD and confidence intervals).

#### Modeling isthmus stem cell fate

To study the fate behavior of the isthmus stem cell population based on the clonal data in normal and perturbed (after DMP-777 treatment) conditions, we use a statistical modeling-based approach adapting the neutral drift dynamics model developed for the intestinal crypt. In this approach, we use quantitative and qualitative features of the data - in this case, the time evolution of the average clone size and the spatial organization of parietal cells around the gland circumference - to develop a simple statistical model of stem cell fate choice based on a minimal number of adjustable parameters. This model then forms a basis for statistical testing of the experimental clone size and Parietal cell number distributions using standard least-squares fitting approach. Details of the modeling framework and the implementation of the statistical analysis (and confidence intervals) are presented in Methods S1.

#### Bulk RNA-seq data analysis

To check the quality of sequenced reads of 100 nt length from the mRNA-seq, reads were imported to FastQC. Based on the FastQC results, 4 nucleotides with low base signal quality at the 3′ end and, if present, poly A-sequences were trimmed from the raw sequences. In addition, random primer sequences (∼12 nucleotides) at the 5′ end were also trimmed according to instructions from the QuantSeq FWD manual. The trimmed reads were aligned to the mouse genome (GRCm38 from Ensemble) using STAR (Dobin et al., 2013) with the default parameter settings. The aligned reads were indexed and sorted by samtools (Li et al., 2009). HTseq was then used to assemble the aligned reads to transcripts and quantify the read counts (Anders et al., 2015). The read counts were normalized across all samples by the trimmed mean of M-values (TMM) normalization method implemented in the R package edgeR (Robinson et al., 2010). Reproducibility between the samples was checked with the principal component analysis (PCA). All samples clustered according to their biological status, confirming good reproducibility between samples for each condition analyzed. To focus on candidate marker genes with relatively high expression, genes with normalized read counts lower than the 90th percentile of the whole read count distribution (∼63 read counts per sample) were excluded from further analysis. To select genes specific to proliferating cells, genes more highly expressed in mVenus+ cells from the *Rosa26-Fucci2a* mouse when compared to E-Cadherin+ cells were defined as those with p < 0.05 from a negative binomial test in DESeq (Anders and Huber, 2010) and a log2 fold-change more than 1. The number of genes meeting these criteria was 150 (Table S2).

As another criterion, microarray data from 5-FU-treated mice was also included in the gene expression analysis. Briefly, 5-FU treatment was employed to deplete proliferating cells. The stomach corpus was collected from 5-FU-treated and untreated mice at 2 d post-treatment and used for microarray analysis. As the replicate number for each condition was only one sample, the ratio of gene expression in the 5-FU-treated sample relative to the untreated sample was calculated and a ratio of less than 1/2 was considered to be a significant change, resulting in the identification of 136 genes that were highly expressed in proliferating cells in the stomach corpus (Table S3).

Following comparison of the two independent gene lists, 54 genes present in both lists were carried forward for further analysis. The Human Protein Atlas (Uhlén et al., 2015) was then used to confirm gene expression in the stomach and used to check the level of protein expression of the 54 genes. Only genes satisfying the following two criteria were considered as plausible candidates: 1) ‘Supported’ or ‘Approved’ in terms of reliability and 2) ‘Medium’ or ‘High’ in terms of expression level. The number of plausible candidate genes was 17. Based on immunostaining results available at the Human Protein Atlas, we further selected Stmn1 and Ki67 as the most likely prospective markers for proliferating cells in the gastric corpus.

#### Single-cell RNA-seq data analysis

Paired-end reads of both Stmn1+ and Pgc+ single cells were mapped to the *Mus musculus* genome (GRCm38) using STAR (v2.5.2b) with default parameters (Dobin et al., 2013). Splice junctions in reads were detected with the help of a GTF file of GRCm38 provided by Ensembl (release 90). Uniquely mapped reads were counted for each gene using htseq-count (v0.7.2) (Anders et al., 2015). We removed poor-quality cells that have greater than 10% reads mapped to mitochondrial-encoded genes and greater than 97% genes expressed below a detection limit of 5 read counts. As a result, 610 Stmn1+ cells and 743 Pgc+ cells passed quality control.

Mapped reads were normalized using pool-based sized factors that were featured in the scran (v1.6.9) package of R (Lun et al., 2016), which allows normalization of sparse single-cell RNA-seq data. The normalized counts were then log2-transformed with a pseudocount count of 1. Highly variable genes were identified using the decomposeVar function of the scran package with options of FDR ≤ 0.05 and biological variability > 0.5. From the log-normalized expression matrix of highly variable genes, we visualized each cell in the 2-dimensional t-SNE plot (van der Maaten and Hinton, 2008) by projecting the expression matrix into the first 50 principal components and then by reducing the dimension of the projected data into 2 based on the t-SNE algorithm implemented in the Rtsne (v0.13) package of R with a perplexity of 50 (van der Maaten, 2014).

For the downstream analysis of Stmn+ single-cell data, we performed a consensus clustering analysis with the SC3 (v1.7.7) package of R (Kiselev et al., 2017). We first estimated the number of clusters using the sc3_estimate_k function and grouped each cell into 9 clusters using highly variable genes. For each cluster, we identified marker genes using the Wilcoxon rank sum test implemented in the Seurat (v2.3.0) package of R with the options of adjusted P value < 0.05 and fold change > 1.5 (Butler et al., 2018).

Pseudotime analysis was conducted by first projecting the log-normalized expression matrix of marker genes of all clusters into 50 diffusion map coordinates using the DiffusionMap (v1.1.0) package of R and then by inferring differentiation trajectories based on the slingshot (v1.0.0) package of R (Street et al., 2018). To obtain two differentiation trajectories, the starting cluster was set to 6 and ending clusters were set to 1 and 2. We also performed two different pseudotime analysis methods to confirm the robustness of our inferred trajectories: monocle (v2.6.4) (Qiu et al., 2017) and destiny (v2.6.2) (Haghverdi et al., 2016). For monocle, we used the expression matrix of marker genes normalized by the scran size factors, reduced the dimension of the matrix to 2 using the method of DDRTree, and ordered cells along the trajectory with the orderCells function of the monocle package of R. For destiny, we calculated diffusion pseudotime (DPT) from the log-normalized expression matrix of marker genes. For each gene, Z-scores of gene expression were plotted using a rolling mean along each of the trajectories with a window size of 5% of cells.

To correct for the effects of cell cycle on cellular heterogeneity in the Stmn1^high^ subpopulation (cluster 4, 5, 6 and 7 in Figure 6A), we first calculated S and G2/M scores of each cell using the CellCycleScoring function of the Seurat (v2.3.0) package of R, and then regressed out the cell cycle scores with the ScaleData function of the Seurat package to obtain a cell cycle corrected expression matrix (Butler et al., 2018). Highly variable genes were identified from the cell cycle corrected expression matrix using the FindVariableGenes function of the Seurat package with the options of x.low.cutoff = 0.0125, x.high.cutoff = 2, and y.cutoff = 2. We identified six subclusters (S1-S6) in the Stmn1^high^ subpopulation using the highly variable genes with the SC3 package. We visualized each cell in the 2-dimensional uniform manifold approximation and projection (UMAP) (Becht et al., 2019) plot using the umap (v0.2.0.0) package of R with default options, where the marker genes of cluster 1 (NL), 2 (PL), and 9 (SL) in Figure 6A were taken as an input gene set, and the second and third UMAP embeddings were used. To estimate the degree of the fate bias of the six subclusters to specific sublineages, we then defined the representative gene expression patterns for neck cells and pit cells by taking the centroids of clusters 1 (NL) and 2 (PL) in Figure 6A, respectively. Next we computed the similarity between each cell in the Stmn1^high^ subpopulation and the representative neck cell and pit cell based on their expression correlation. To refine the lineage relationships between the six subclusters, we again performed the pseudotime analysis for S1-S6 using Slingshot. As a result, two differentiation trajectories inferred by the slingshot package (starting cluster set as S4 and ending clusters as S3 and S6) were projected into the UMAP plot. The six subclusters were merged into three groups using hierarchical clustering with the Spearman rank correlation based distance implemented in the amap (v0.8-16) package of R: S4 and S5 for stem-like group; S1, S2 and S3 for pit cell progenitor group; S6 for neck cell progenitor group. For each group, we identified marker genes using the Wilcoxon rank sum test implemented in the Seurat package with the options of adjusted P value < 0.05 and fold change > 1.5. The marker gene set were clustered along each trajectory with k-mean clustering (k = 5) implemented in the pheatmap (v1.0.12) package of R after smoothing the cell cycle corrected expression of genes along the trajectories with the loess function of R (Table S4).

### Data and Code Availability

The accession numbers for the bulk RNA-seq data from the *Rosa26-Fucci2a* model and single-cell RNA-seq data are ArrayExpress: E-MTAB-6850 and E-MTAB-6879, respectively.

The raw datasets/codes generated during our modeling and single-cell RNA-seq analysis can be found in the following repository: https://github.com/scg-dgist/CELL-STEM-CELL-isthmus-stem-cells.

## Supporting Citations

The following references appear in the Supplemental Information: Gillespie, 1977, Roff, 2006.

## Supplemental Information

Document S1. Figures S1–S7, Methods S1, and Table S1

Table S2. List of Genes More Highly Expressed in mVenus+ Cells from the Rosa26-Fucci2a Mouse, Related to Figure 5

Table S3. List of Genes Depleted in the Stomach Corpus upon 5-FU Treatment, Related to Figure 5

Table S4. List of Genes Showing Distinct Expression Patterns along the Pseudotime Orders from IsthSC toward Neck or Pit Cell Lineage, Related to Figure 6

Document S2. Article plus Supplemental Information
