# Supplementary material for: Defining the Identity and Dynamics of Adult Gastric Isthmus Stem Cells
Source: Cell Stem Cell. 2019 Sep 5;25(3):342–356.e7. doi: 10.1016/j.stem.2019.07.008 (PMC6739486; doi:10.1016/j.stem.2019.07.008)
Supplement: Document S1. Figures S1–S7, Methods S1, and Table S1 [file mmc1.pdf]

Cell Stem Cell, Volume *25*

Supplemental Information

Defining the Identity and Dynamics of Adult Gastric Isthmus Stem Cells

Seungmin Han, Juergen Fink, David J. Jörg, Eunmin Lee, Min Kyu Yum, Lemonia Chatzeli, Sebastian R. Merker, Manon Josserand, Teodora Trendafilova, Amanda Andersson-Rolf, Catherine Dabrowska, Hyunki Kim, Ronald Naumann, Ji-Hyun Lee, Nobuo Sasaki, Richard Lester Mort, Onur Basak, Hans Clevers, Daniel E. Stange, Anna Philpott, Jong Kyoung Kim, Benjamin D. Simons, and Bon-Kyoung Koo

Figure S1

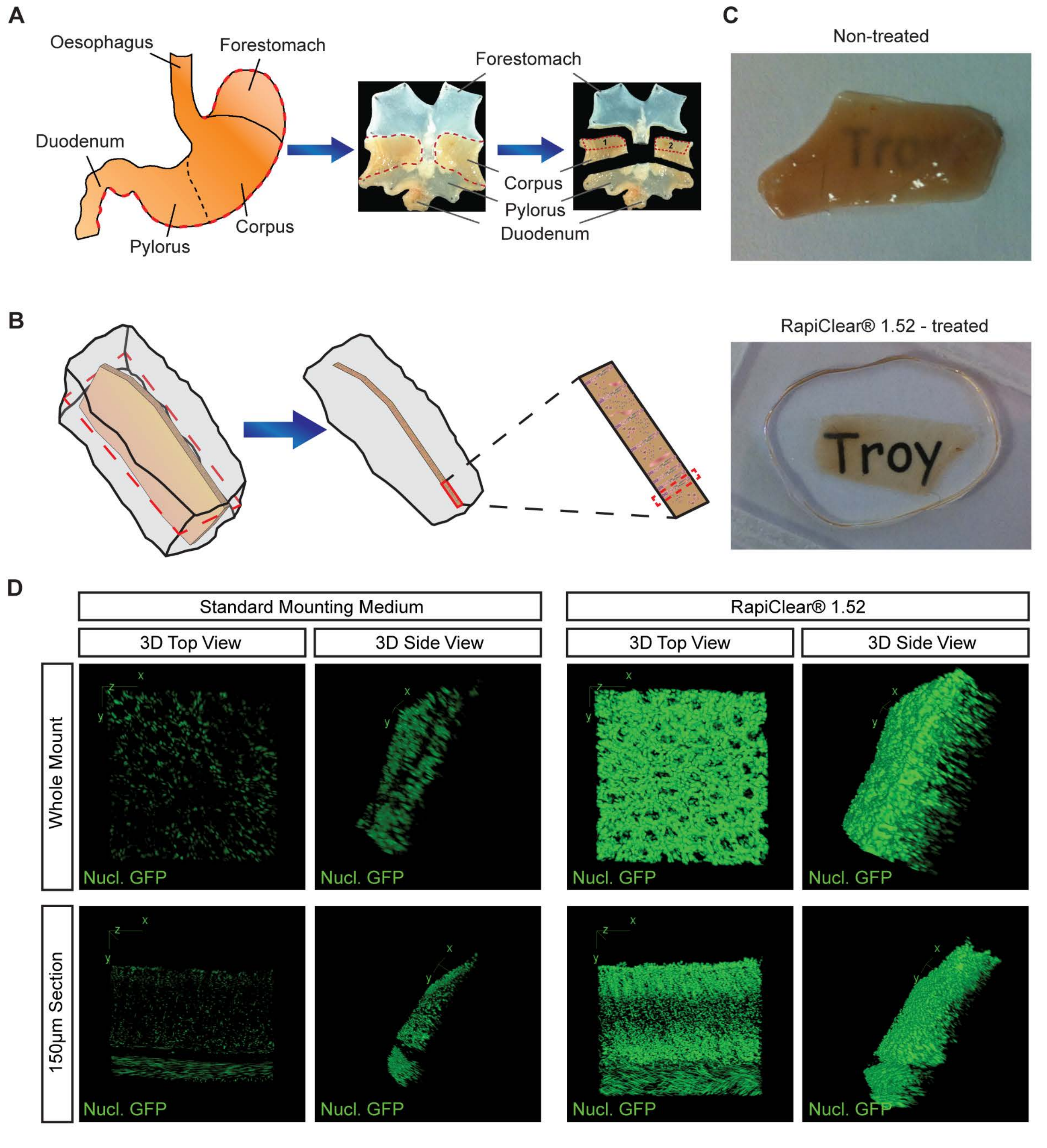
A
Oesophagus
Forestomach
Duodenum
Pylorus
Corpus
Forestomach
Corpus
Pylorus
Duodenum
1
2
B
C
Non-treated
Troy
RapiClear® 1.52 - treated
Troy
D
Standard Mounting Medium
RapiClear® 1.52
3D Top View
3D Side View
3D Top View
3D Side View
Whole Mount
150µm Section
Nucl. GFP
Nucl. GFP
Nucl. GFP
Nucl. GFP
Nucl. GFP
Nucl. GFP
Nucl. GFP
Nucl. GFP

Figure S1. Stomach clearing and deep tissue imaging using RapiClear 1.52, related to Figure 1.

(A) Anatomy of the mouse stomach before (left panel) and after spreading and fixation (middle panel). Right panel: dissection and selection of corpus for subsequent agarose embedding. #1 and #2 are both embedded to represent both sides of the stomach. Note: the selected corpus region is smaller than the actual size of the corpus to ensure corpus gland identity during analysis of labeling.

(B) Schematic of corpus agarose embedding (one of the two corpus pieces shown in (A) is depicted). Entire glands can be obtained by sectioning to generate 150 µm-thick sections.

(C) Stomach tissue is rendered transparent after 4 h incubation in RapiClear 1.52.

(D) Deep tissue imaging of nuclear H2B-GFP, in combination with matched refractive index optical tissue clearing. Whole mount and 150 µm-thick sections of a mouse stomach expressing nuclear-localized GFP were imaged by conventional single photon microscopy with the exact same laser and confocal settings between samples mounted in RapiClear 1.52 and samples mounted in standard mounting medium. Our deep tissue imaging combined with tissue clearing enables us to recover the precise location of individually-labeled cells to assess both the horizontal and vertical intra-glandular clonal expansion patterns.

Figure S2

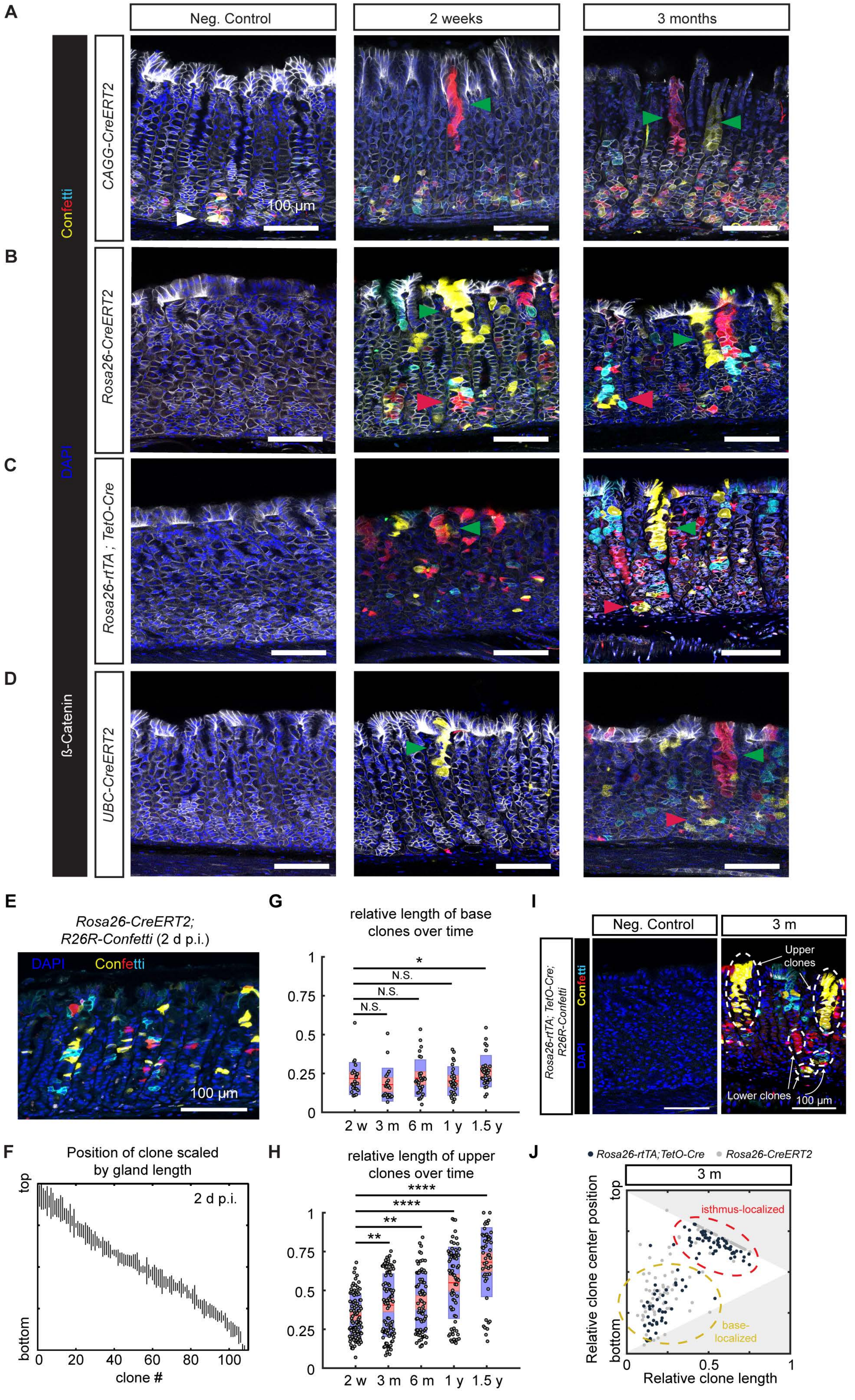

A
B
C
D
Neg. Control
2 weeks
3 months
CAGG-CreERT2
Rosa26-CreERT2
Rosa26-rtTA ; TetO-Cre
UBC-CreERT2
Confetti
DAPI
ß-Catenin
100 µm
E
Rosa26-CreERT2;
R26R-Confetti (2 d p.i.)
DAPI
Confetti
100 µm
F
Position of clone scaled
by gland length
2 d p.i.
top
bottom
clone #
G
relative length of base
clones over time
*
N.S.
N.S.
N.S.
2 w
3 m
6 m
1 y
1.5 y
H
relative length of upper
clones over time
****
****
**
**
I
Neg. Control
3 m
Rosa26-rtTA; TetO-Cre;
R26R-Confetti
Upper
clones
Lower clones
J
Rosa26-rtTA;TetO-Cre
Rosa26-CreERT2
3 m
isthmus-localized
base-
localized
Relative clone center position
Relative clone length

Figure S2. Screening of available ubiquitous, inducible Cre recombinase mouse models crossed with the *R26R-Confetti* reporter line, related to Figure 1.

(A-D) Four available mouse models expressing tamoxifen- or doxycycline-inducible Cre recombinase under the control of the indicated ubiquitous promoters were crossed with the *R26R-Confetti* reporter line (A: *CAGG-CreERT2*, B: *Rosa26-CreERT2*, C: *Rosa26-rtTA;TetO-Cre*, D: *UBC-CreERT2*) to find the optimal line for clonal density labeling. 150 µm-thick mouse corpus sections were stained for β-Catenin as a membrane marker for better gland visualization (Grey). Nuclei were stained with DAPI (Blue). Analysis of uninduced mice demonstrated leaky Cre activity in the base of the corpus glands in *CAGG-CreERT2* mice (A; white arrow heads). *Rosa26-CreERT2* and *Rosa26-rtTA;TetO-Cre* mice showed no evidence of recombination at the confetti locus in the absence of tamoxifen or doxycycline administration, respectively (B, C). Induction of Cre recombinase resulted in labeled clonal expansion in the isthmus region by 2 w post-administration in all mouse models tested (A, B, C, D; green arrow heads). Isthmus-derived clones remained restricted to the pit, isthmus and neck region and did not reach the base of corpus glands up to 3 m post-induction (A, B, C, D). Cells located in the base of corpus glands were successfully labeled at 2 w post-induction to varying degrees in all mouse models tested and clones remained small up to 3 m post-induction (red arrow heads). *Rosa26-rtTA;tetO-Cre;R26R-Confetti* and *UBC-CreERT2;R26R-Confetti* lines were not optimal due to their additional transgene requirement and lower labeling efficiency, respectively (C and D). Thus, we chose to focus on the *Rosa26-CreERT2;R26R-Confetti* mouse line in all subsequent experiments. Yellow: EYFP; Red: tdimer2; Cyan: mCerulean; Grey: β-Catenin; Blue: DAPI. Scale bars: 100 µm.

(E) A representative image showing random labeling in the stomach corpus gland of *Rosa26-CreERT2;R26R-Confetti* mice at 2 d post-induction (p.i.). Yellow: EYFP; Red: tdimer2; Cyan: mCerulean; Grey: β-Catenin; Blue: DAPI. Scale bar: 100 µm.

(F) Corresponding plot of clone length and position scaled by gland length along the axis of the gland shows the random labeling in the stomach corpus gland at the same 2 d time-point. N=108 clones pooled from 2 mice were analyzed.

(G, H) The relative lengths of the base clones (G) and upper clones (H) over time in *Rosa26-CreERT2;R26R-Confetti*. A clone is considered to be a base clone if the relative position of the clone center is below 0.3. Otherwise, the clone is considered to be an upper clone. Note that the positions of the gland bottom and top are 0 and 1, respectively. N=114 clones (2w), 109 clones (3m), 104 clones (6m), 99 clones (1y) and 82 clones (1.5y) were pooled from 2 mice per time-point. Red line: mean; red-shaded box: 95% CI; blue-shaded box: SD. N.S.: statistically not significant (p-value > 0.1); *p-value < 0.05; **p-value < 0.01; ****p-value < 1e-9 (two-sample Kolmogorov-Smirnov test used).

(I) Representative image of *Rosa26-rtTA;TetO-Cre;R26R-Confetti* mouse stomach corpus glands showing the maintenance of the two compartments at 3 m following induction with doxycycline. Yellow: EYFP; Red: tdimer2; Cyan: mCerulean; Blue: DAPI. Scale bars: 100 µm.

(J) Scatter plot of relative (vertical) clone length and clone center position in *Rosa26-rtTA;TetO-Cre; R26R-Confetti* line at the same time-point as (I). Clone characteristics illustrate that a separation over time into larger clones (inside red dotted ellipse) in the isthmus-pit region and smaller clones (inside yellow dotted ellipse) in the base region of corpus glands is maintained even in the doxycycline-inducible *Rosa26-rtTA;TetO-Cre;R26R-Confetti* line, supporting the existence of the two compartments regardless of any tamoxifen-induced damage. Grey dots: relative clone length and center positions in *Rosa26-CreERT2;R26R-Confetti* at 3 m post-labelling by tamoxifen. N=109 clones

pooled from 2 mice for the *Rosa26-CreERT2;R26R-Confetti* model and N=104 clones from 1 mouse for the *Rosa26-rtTA;TetO-Cre;R26R-Confetti* model were analyzed.

Figure S3

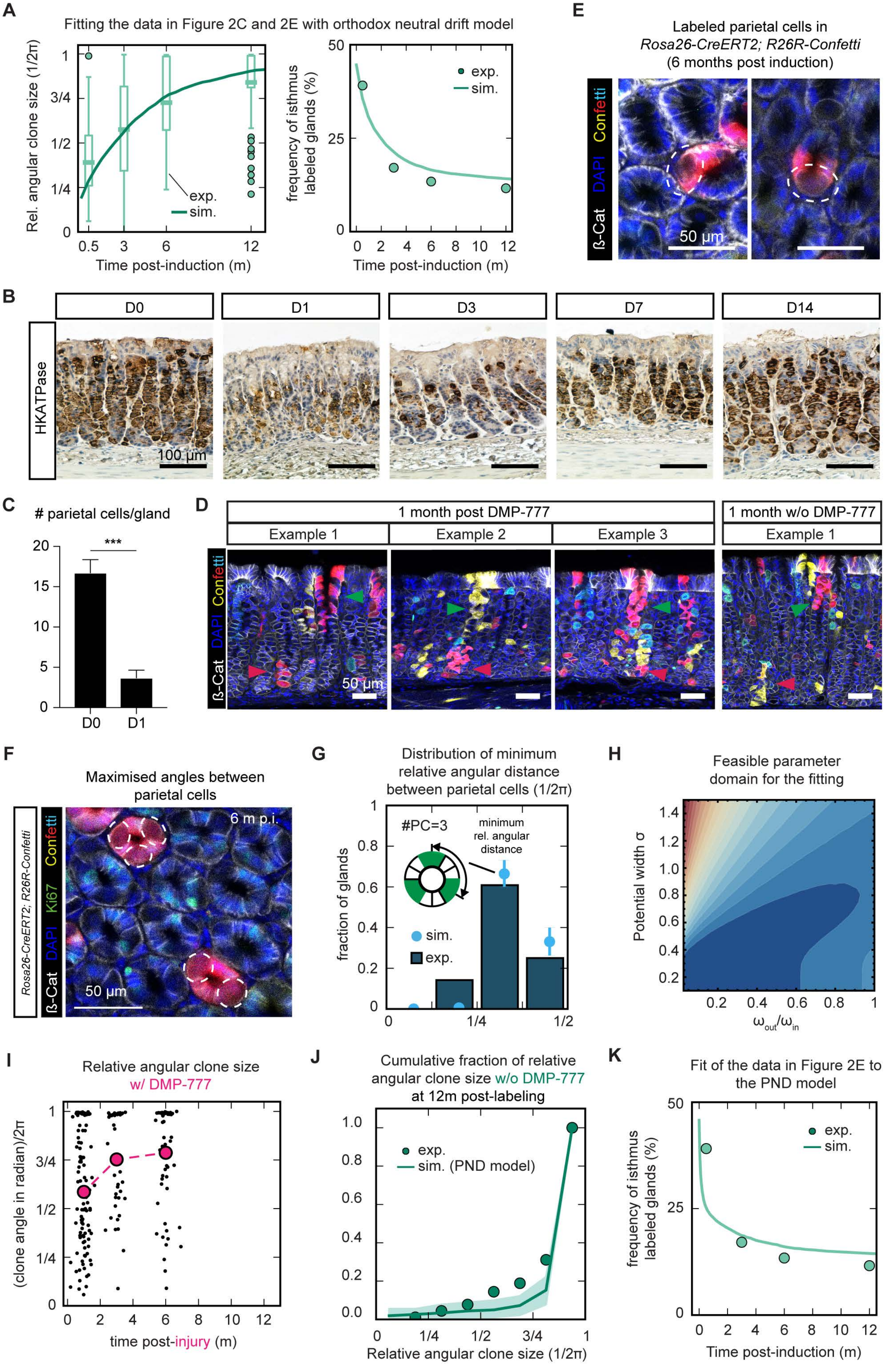
A
Fitting the data in Figure 2C and 2E with orthodox neutral drift model
Rel. angular clone size (1/2π)
exp.
sim.
Time post-induction (m)
frequency of isthmus labeled glands (%)
E
Labeled parietal cells in Rosa26-CreERT2; R26R-Confetti (6 months post induction)
ß-Cat DAPI Confetti
50 µm
B
D0
D1
D3
D7
D14
HKATPase
100 µm
C
parietal cells/gland
***
D0
D1
D
1 month post DMP-777
1 month w/o DMP-777
Example 1
Example 2
Example 3
Example 1
50 µm
F
Maximised angles between parietal cells
Rosa26-CreERT2; R26R-Confetti
ß-Cat DAPI Ki67 Confetti
6 m p.i.
50 µm
G
Distribution of minimum relative angular distance between parietal cells (1/2π)
#PC=3
minimum rel. angular distance
sim.
exp.
fraction of glands
H
Feasible parameter domain for the fitting
Potential width σ
ω_{out}/ω_{in}
I
Relative angular clone size w/ DMP-777
(clone angle in radian)/2π
time post-injury (m)
J
Cumulative fraction of relative angular clone size w/o DMP-777 at 12m post-labeling
exp.
sim. (PND model)
Relative angular clone size (1/2π)
K
Fit of the data in Figure 2E to the PND model
exp.
sim.
frequency of isthmus labeled glands (%)
Time post-induction (m)

Figure S3. Parietal cells establish a barrier inhibiting the lateral expansion of isthmus clones, related to Figure 2, 3, and 4.

(A) Fit of simulated data to experimentally-derived measurements of relative angular clone size and survival curve of isthmus-labeled glands. The box plots with medians in the centers (left graph) and green dots (right graph) represent experimental data. The solid line is the fit of the data generated using the orthodox neutral drift model. The same experimental data in Figures 2C and 2E were used.

(B) Immunohistochemical staining for HKATPase counterstained with Mayer's Haematoxylin at multiple time-points following DMP-777 treatment of mouse stomach corpus sections. Scale bars: 100µm.

(C) Quantification of HKATPase+ cells (viz. parietal cells) per gland in the control and at 1 d post-DMP-777 treatment. Data are presented as mean $\pm$ SD. *** $p < 0.0001$ (t-test). Parietal cells were counted from N=15 glands of 1 mouse at each time-point.

(D) Representative confocal images of stomach corpus from *Rosa26-CreERT2;R26R-Confetti* mice at 1 m post-labeling with and without DMP-777 administration. Upper and lower clones are indicated by arrow heads (green: clones in the isthmus-pit; red: clones in the base). Yellow: EYFP; Red: tdimer2; Cyan: mCerulean; Grey: β-Catenin; Blue: DAPI. Scale bars: 50µm.

(E) Representative whole mount image of stomach corpus tissue from *Rosa26-CreERT2;R26R-Confetti* mice at 6 m post-tamoxifen administration. Parietal cells (indicated by dotted white lines) are labeled with the same confetti color as neighboring isthmus cells. Red: tdimer2; Grey: β-Catenin; Blue: DAPI. Scale bars: 50 µm.

(F) Representative whole mount image of stomach corpus tissue from *Rosa26-CreERT2;R26R-Confetti* mice at 6 m post-tamoxifen administration. Parietal cells (indicated by dotted white lines) show a maximum angular distribution within the circumference of the glands. Red: tdimer2; Green: Ki67; Grey: β-Catenin; Blue: DAPI. Scale bar: 50 µm.

(G) Distribution of minimum relative angular distance between parietal cells for 3 parietal cells per gland isthmus region. The minimum angular distance is the smallest angle between a pair of PCs. Sky blue dots and lines denote means and SD, respectively, derived from model simulations and dark blue bars indicate experimental data. The modal distance is around 120°. N=120 glands were analyzed.

(H) Residual values from the standard least-square approach in the parameter space of (σ, r). σ is the characteristic length scale (in segments) over which the probability drops to zero outside an existing barrier segment. $r = \omega_{\text{out}}/\omega_{\text{in}}$ represents the ratio of the loss rate and reference birth rate of barrier segments. The parameter combination was restricted to the regions in which the residual d was not larger than the 70-fold minimum of d (dark blue region), which ensured that the region did not exceed the upper parameter boundaries.

(I) Relative angular clone size of isthmus-derived clones at 1 m, 3 m and 6 m post-induction following administration of DMP-777. Black dots represent the measurements from individual clones. Red dots are the average values of clone size for each time-point, with the dotted line linking the average values to show the trend over time. N=99 clones (1m), 45 clones (3m) and 61 clones (6m) were pooled from 2-3 mice per time-point.

(J) Cumulative fraction of the relative angular clone size in control samples at 12 m post-induction. Dots indicate the frequency as determined from experimental data. N=90 clones were pooled from 3 mice at 12m post-induction. The line indicates the fit of simulated results from the punctuated neutral

drift (PND) model with incorporation of PCs as physical barriers and only different initial conditions. Shading displays the 95% CI around the fit of simulated data.

(K) Fit of simulated data to the survival curve for isthmus-labeled glands. Dots indicate the frequency as determined from experimental data used in Figure 2E. The line indicates the fit of simulated results from the PND model.

Figure S4

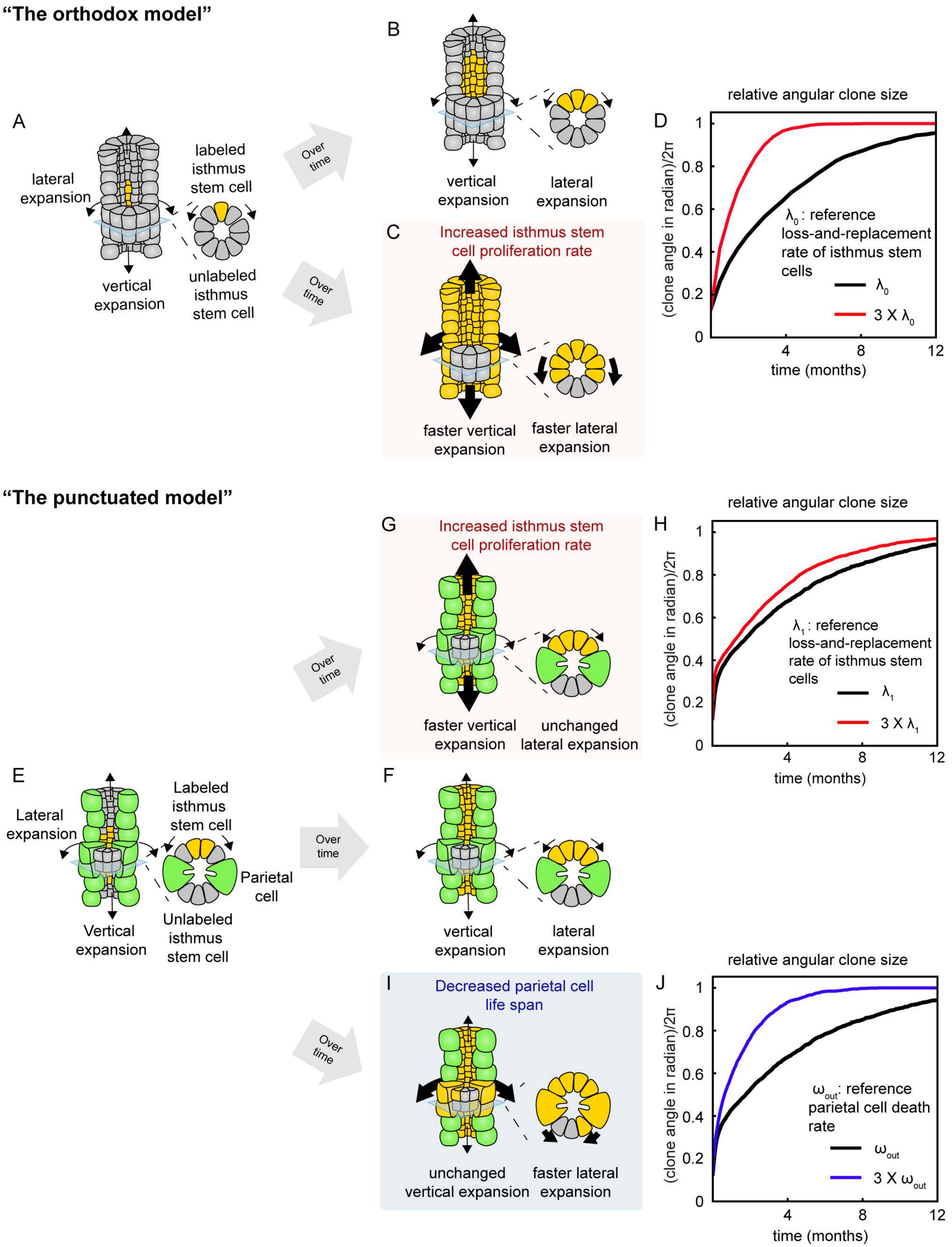

"The orthodox model"
A
lateral expansion
labeled isthmus stem cell
vertical expansion
unlabeled isthmus stem cell
Over time
Over time
B
vertical expansion
lateral expansion
C
Increased isthmus stem cell proliferation rate
faster vertical expansion
faster lateral expansion
D
relative angular clone size
(clone angle in radian)/2π
λ_0: reference loss-and-replacement rate of isthmus stem cells
λ_0
3 X λ_0
time (months)
"The punctuated model"
G
Increased isthmus stem cell proliferation rate
faster vertical expansion
unchanged lateral expansion
Over time
H
relative angular clone size
(clone angle in radian)/2π
λ_1: reference loss-and-replacement rate of isthmus stem cells
λ_1
3 X λ_1
time (months)
E
Lateral expansion
Labeled isthmus stem cell
Parietal cell
Vertical expansion
Unlabeled isthmus stem cell
Over time
F
vertical expansion
lateral expansion
I
Decreased parietal cell life span
unchanged vertical expansion
faster lateral expansion
Over time
J
relative angular clone size
(clone angle in radian)/2π
ω_{out}: reference parietal cell death rate
ω_{out}
3 X ω_{out}
time (months)

Figure S4. The orthodox neutral drift model and punctuated neutral drift model produce different predictions about the effect of perturbed parameters on vertical and lateral clone expansion, related to Figure 4.

(A) Hypothetical gland structure without any physical barriers that follows the orthodox model of stem cell loss-and-replacement in glandular epithelia. This schematic corresponds to the clonal labeling state (by yellow) soon after clone induction (e.g. 2 d post-induction).

(B and C) These schematics correspond to the labeling state at a few months post-labelling under different conditions. In (C), the IsthSC proliferation rate is faster than that in (B), resulting in an increased loss-and-replacement rate.

(D) Time evolution of the average relative angular clone size depending on different loss-and-replacement rates (λ) of IsthSCs in (B) (black line) and (C) (red line). Black line: reference value of λ (= λ_0) from data fitting with the orthodox model during homeostasis; red line: 3 x λ_0.

(E) Gland structure around the isthmus region of the gastric corpus gland. Green: parietal cells. This corresponds to the clonal labeling state (by yellow) soon after clone induction (e.g. 2 d post-induction).

(F, G and I). These schematics correspond to the labeling state at a few months post-labelling under different conditions. With reference to (F) as the control case, (G) corresponds to increased proliferation of IsthSCs, whereas (I) corresponds to decreased parietal cell life span. (G) and (I) denote accelerated vertical and lateral expansion under the aforementioned conditions, respectively.

(H) The evolution of averaged relative angular clone size depending on different loss-and-replacement rates (λ) of IsthSCs in (F) (black line) and (G) (red line). Black line: reference value of λ (= λ_1) from data fitting with the punctuated model; red line: 3 x λ_1

(J) The evolution of averaged relative angular clone size depending on different rates (ω) of parietal cell death in the isthmus section in (F) (black line) and (I) (blue line). Black line: reference value of ω (= ω_0) from data fitting with the punctuated model; blue line: 3 X ω_0.

Figure S5

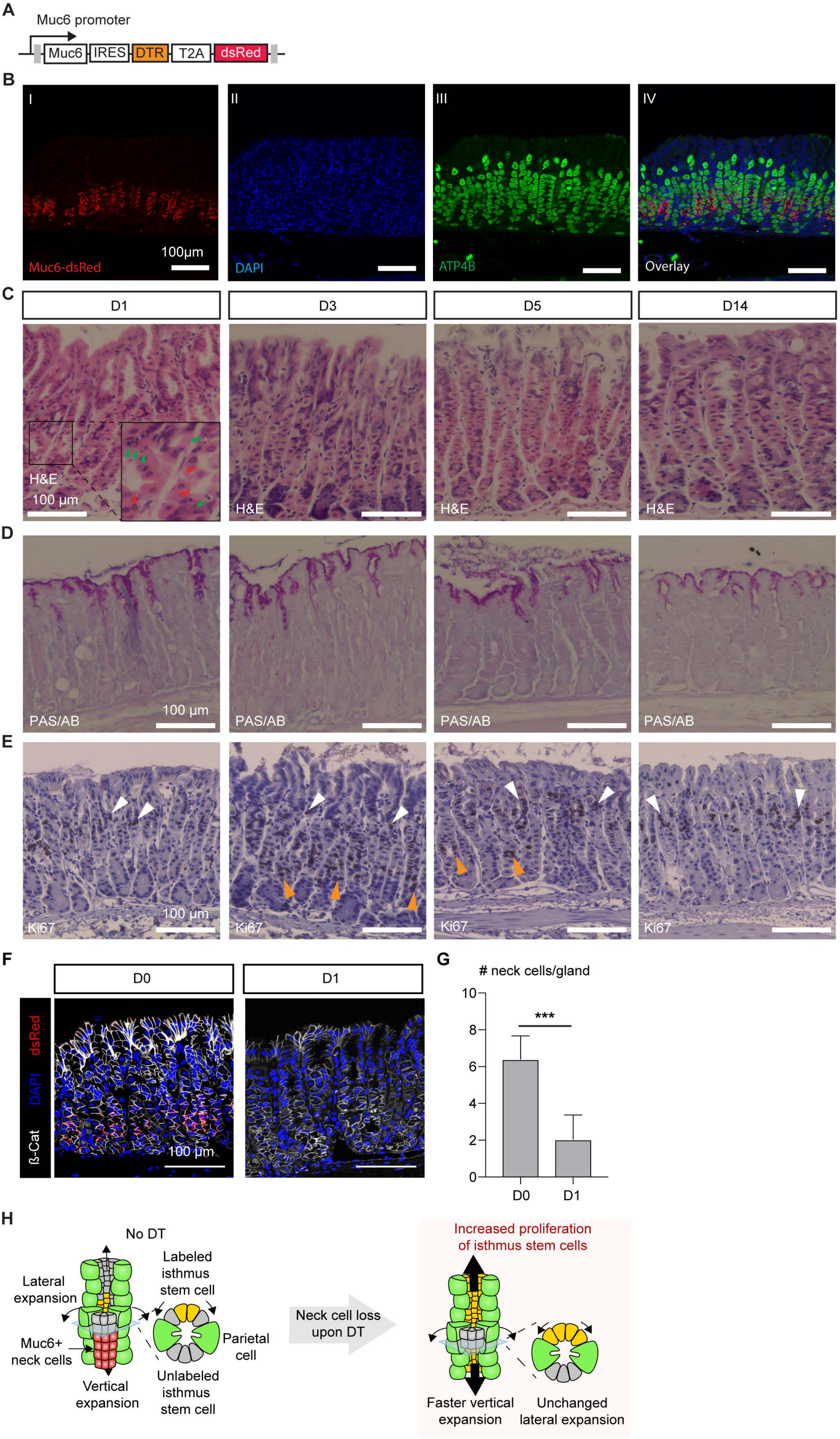
A
Muc6 promoter
Muc6
IRES
DTR
T2A
dsRed
B
I
II
III
IV
100µm
Muc6-dsRed
DAPI
ATP4B
Overlay
C
D1
D3
D5
D14
H&E
100 µm
D
PAS/AB
100 µm
E
Ki67
100 µm
F
D0
D1
dsRed
DAPI
ß-Cat
100 µm
G
neck cells/gland
10
8
6
4
2
0
***
D0
D1
H
No DT
Lateral expansion
Labeled isthmus stem cell
Muc6+ neck cells
Parietal cell
Unlabeled isthmus stem cell
Vertical expansion
Neck cell loss upon DT
Increased proliferation of isthmus stem cells
Faster vertical expansion
Unchanged lateral expansion

Figure S5. Injury experiments using *Muc6-DTR;Rosa26-CreERT2;R26R-Confetti* mice confirm the punctuated neutral drift model, related to Figure 4.

(A) Schematic of the *Muc6-IRES-DTR-T2A-dsRed (abbr. Muc6-DTR)* construct.

(B) Confocal image of 100 µm-thick section of *Muc6-DTR* mouse stomach corpus. Red: Muc6-dsRed; Blue: DAPI; Green: ATP4B. Scale bars: 100 µm.

(C-E) *Muc6-DTR* mice were injected with 1 ng diphtheria toxin (DT) per 1 g of mouse body weight. Mouse stomach corpus samples were collected at d1, d3, d5 and d14 post-DT administration. H&E staining (C) shows the presence of apoptotic bodies (inset, green arrowheads) as well as lesions (inset, red arrowheads) in the neck region of corpus glands on d1 post-DT administration. PAS/AB staining (D) shows no significant difference in mucus secretion. Ki67 staining (E) shows increased numbers of Ki67+ cells in the gland neck at 3d and 5d post-mucus neck cell depletion. Orange arrowheads indicate base/neck-located proliferative cells. White arrowheads indicate isthmus-located proliferative cells. Scale bars: 100 µm.

(F) Confocal image of *Muc6-IRES-DTR-T2A-dsRed* mouse stomach corpus sections under control condition and at d1 post-DT treatment. Blue: DAPI; Grey: β-Catenin; Red: dsRed. Scale bars: 100 µm.

(G) Quantification of dsRed+ cells (viz. neck cells) per gland in control glands and at d1 post-DT treatment. Data are presented as mean $\pm$ SD. *** $p < 0.0001$ (t-test). Neck cells were counted from N=20 glands of 1 mouse at each time-point.

(H) Schematic of *Muc6-DTR* experiments. During homeostasis (no DT), Muc6+ neck cells (highlighted in red) are located close to the IsthSCs. Upon DT treatment, the neck cells are depleted while preserving parietal cells intact. This injury may directly stimulate the IsthSCs adjacent to the damaged neck cells, which would increase IsthSC proliferation. Enhanced IsthSC proliferation in the presence of parietal cells causes faster clone expansion in the vertical direction, but not in the lateral direction.

Figure S6

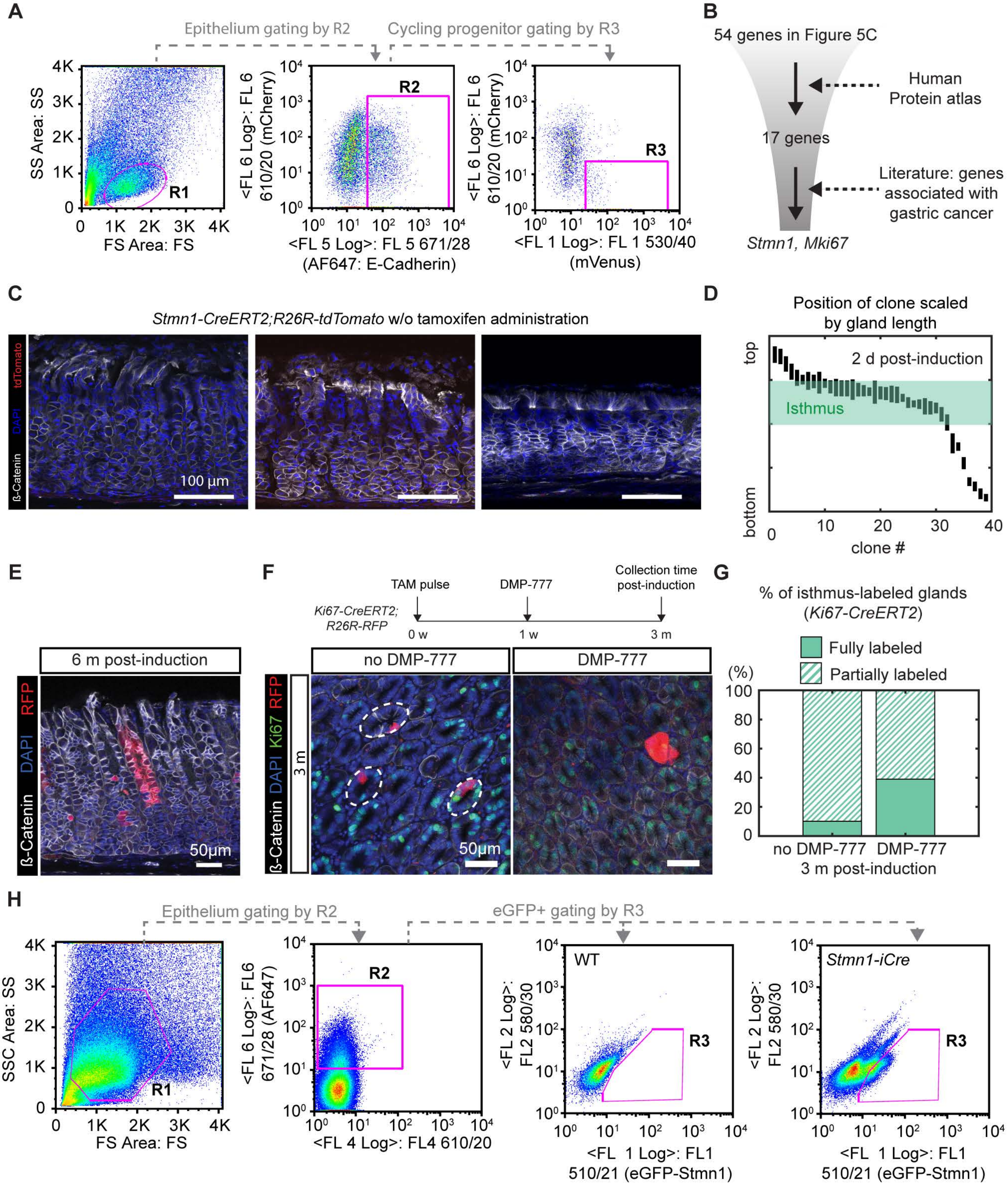

A
Epithelium gating by R2
Cycling progenitor gating by R3
SS Area: SS
FS Area: FS
R1
R2
R3
<FL 6 Log>: FL 6 610/20 (mCherry)
<FL 5 Log>: FL 5 671/28 (AF647: E-Cadherin)
<FL 1 Log>: FL 1 530/40 (mVenus)
B
54 genes in Figure 5C
Human Protein atlas
17 genes
Literature: genes associated with gastric cancer
Stmn1, Mki67
C
Stmn1-CreERT2;R26R-tdTomato w/o tamoxifen administration
tdTomato
DAPI
ß-Catenin
100 µm
D
Position of clone scaled by gland length
top
bottom
2 d post-induction
Isthmus
clone #
E
6 m post-induction
RFP
DAPI
ß-Catenin
50µm
F
Ki67-CreERT2; R26R-RFP
TAM pulse
DMP-777
Collection time post-induction
0 w
1 w
3 m
no DMP-777
DMP-777
ß-Catenin DAPI Ki67 RFP
3 m
50µm
G
% of isthmus-labeled glands (Ki67-CreERT2)
Fully labeled
Partially labeled
(%)
no DMP-777
DMP-777
3 m post-induction
H
Epithelium gating by R2
eGFP+ gating by R3
SSC Area: SS
FS Area: FS
R1
R2
R3
<FL 6 Log>: FL6 671/28 (AF647)
<FL 4 Log>: FL4 610/20
WT
Stmn1-iCre
<FL 2 Log>: FL2 580/30
<FL 1 Log>: FL1 510/21 (eGFP-Stmn1)

Figure S6. Rapidly cycling isthmus progenitors can maintain long-term self-renewal potential, related to Figure 5.

(A) Sorting strategy to isolate proliferative isthmus cells from the stomach corpus of *Rosa26-Fucci2a* mice by FACS. R1: gating for the viable cell population. R2: gating for the stomach corpus epithelium based on the expression of E-Cadherin. R3: gating for proliferating cells (mVenus+/mCherry-).

(B) Schematic outlining the selection of *Stmn1* as a candidate marker for IsthSCs.

(C) Representative confocal images of the mouse stomach corpus glands of *Stmn1-P2A-eGFP-IRES-CreERT2* (*Stmn1-iCre*) mice without tamoxifen administration. Red: tdTomato; Grey: β-Catenin; Blue: DAPI. Scale bars: 100 μm.

(D) Position of clones scaled by gland length. Clone position analysis shows the majority of Stmn1-derived clones are located in the isthmus/pit region at 2 d post-induction. N=39 clones from 2 mice were analyzed.

(E) Representative image from confocal z-stack images of *Ki67-CreERT2;R26R-RFP (Ki67-iCre)* mouse corpus sections at 6 m post-induction. Red: RFP; Grey: β-Catenin; Blue: DAPI. Scale bar: 50 μm.

(F) Representative whole mount images of stomach corpus of *Ki67-iCre* mice at 3m following DMP-777 treatment. Red: RFP; green: Ki67; grey: β-Catenin; blue: DAPI. Scale bar: 50 μm.

(G) The percentage of partially-labeled glands in untreated and DMP-777-treated samples was quantified at 3 m post-induction. n=3 mice for each condition. N=65 glands and 54 glands, pooled from 3 mice per condition, were analyzed in untreated and DMP-777-treated samples, respectively.

(H) Sorting strategy to isolate Stmn1+ cells from the stomach corpus of *Stmn1-iCre* mice by FACS. R1: gating for the viable cell population. R2: gating for the stomach corpus epithelium based on the expression of E-Cadherin. R3: gating for Stmn1+ cells based on the expression of eGFP.

Figure S7

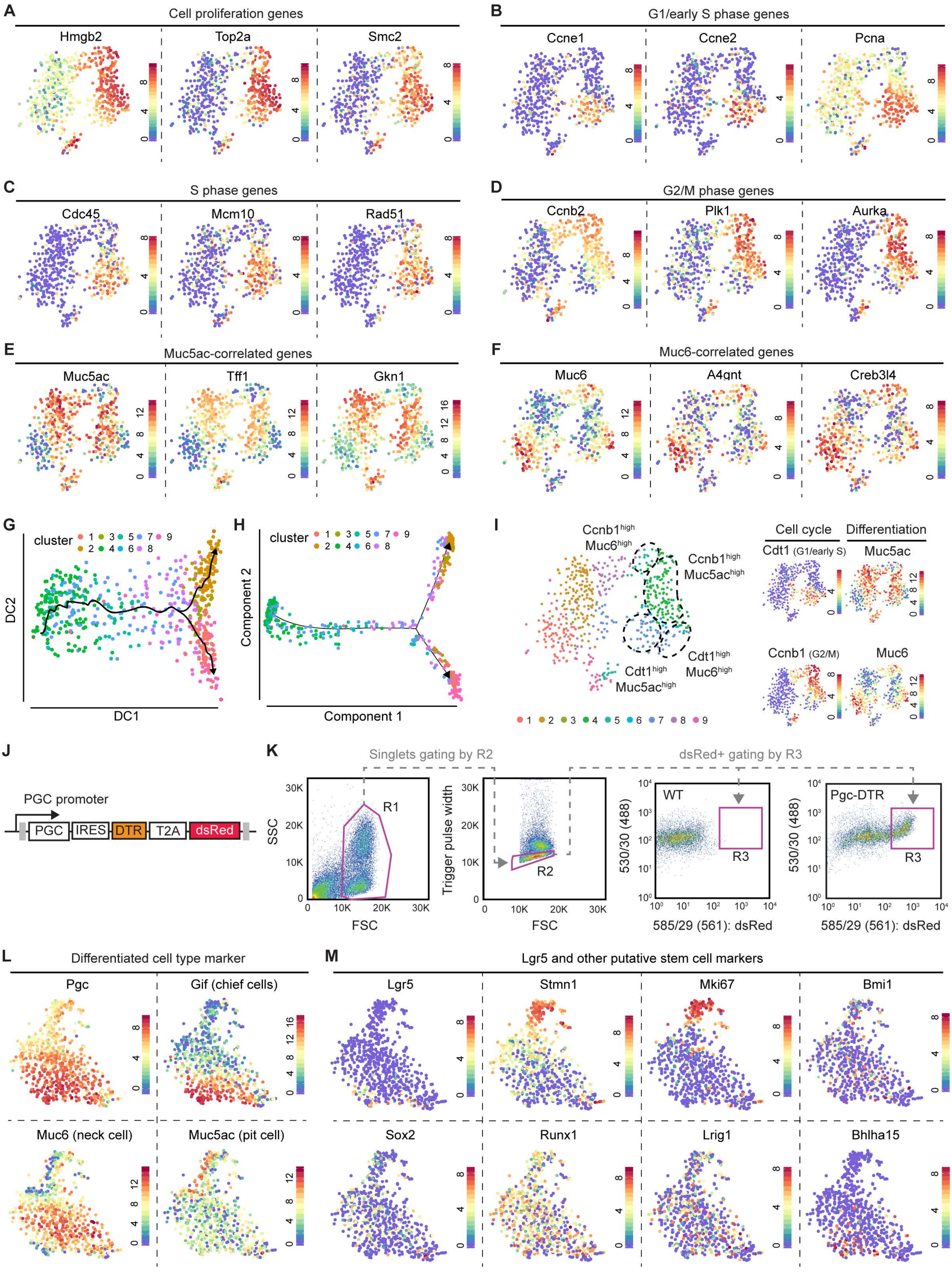

A
Cell proliferation genes
Hmgb2
Top2a
Smc2
B
G1/early S phase genes
Ccne1
Ccne2
Pcna
C
S phase genes
Cdc45
Mcm10
Rad51
D
G2/M phase genes
Ccnb2
Plk1
Aurka
E
Muc5ac-correlated genes
Muc5ac
Tff1
Gkn1
F
Muc6-correlated genes
Muc6
A4gnt
Creb3l4
G
cluster
DC2
DC1
H
cluster
Component 2
Component 1
I
Ccnb1high Muc6high
Ccnb1high Muc5achigh
Cdt1high Muc6high
Cdt1high Muc5achigh
Cell cycle
Differentiation
Cdt1 (G1/early S)
Muc5ac
Ccnb1 (G2/M)
Muc6
J
PGC promoter
PGC
IRES
DTR
T2A
dsRed
K
Singlets gating by R2
dsRed+ gating by R3
SSC
FSC
R1
Trigger pulse width
R2
WT
Pgc-DTR
R3
530/30 (488)
585/29 (561): dsRed
L
Differentiated cell type marker
Pgc
Gif (chief cells)
Muc6 (neck cell)
Muc5ac (pit cell)
M
Lgr5 and other putative stem cell markers
Lgr5
Stmn1
Mki67
Bmi1
Sox2
Runx1
Lrig1
Bhlha15

Figure S7. Single-cell RNA-seq analysis defines the molecular signature and lineage relationships of the cycling IsthSC pool, related to Figure 6 and 7.

(A-D) Gene expression overlaid onto the t-SNE map derived from Stmn1+ single-cell RNA-seq data for representative genes for cell proliferation (A), G1/early S phase (B), S phase (C) and G2/M phase (D). The color bar indicates log2-transformed normalized read counts.

(E, F) Gene expression overlaid onto the t-SNE map derived from Stmn1+ single-cell RNA-seq data for representative genes for pit cell (E) and neck cell (F) lineages. The color bar indicates log2-transformed normalized read counts.

(G, H) Pseudotime analysis of Stmn1+ single-cell RNA-seq data using destiny (Haghverdi et al., 2016) (G) and monocle (Qiu et al., 2017) (H). All three methods used (slingshot, destiny and monocle) result in similar pseudotime trajectories, confirming the robustness of the differentiation trajectory inferred from the Stmn1+ single-cell RNA-seq data.

(I) Gene expression overlaid onto the t-SNE map for representative genes for cell proliferation, cell cycle and differentiation. The whole population of the t-SNE map was partitioned into Stmn1 high (right) and low (left) subpopulations. This partitioning was consistent with segregation based on the level of Mki67 expression (high and low populations). The $Stmn1^{high}$ subpopulation was mainly segregated along the different cell cycle phases into G1/early S (lower) and G2/M (upper) populations. The G1/early S and G2/M populations were again segregated along differentiation markers into $Muc5ac^{high}$ cells and $Muc6^{high}$ cells. The color bar indicates log2-transformed normalized read counts.

(J) Schematic of the *Pgc-IRES-DTR-T2A-dsRed (abbr. Pgc-DTR)* construct.

(K) Sorting strategy to isolate Pgc+ cells from the stomach corpus of *Pgc-DTR* knock-in mice by FACS. R1: gating for the viable cell population. R2: gating for singlets. R3: gating for Pgc+ cells based on expression of dsRed.

(L) Gene expression overlaid onto the t-SNE map derived from Pgc+ single-cell RNA-seq data for marker genes for differentiated cell types of the mouse stomach corpus. The color bar indicates log2-transformed normalized read counts.

(M) Gene expression overlaid onto the t-SNE map derived from Pgc+ single-cell RNA-seq data for Lgr5 and putative isthmus stem cell marker genes in the mouse gastric corpus glands. The reported marker genes Sox2, Runx1, Lrig1, Mist1 (Bhlha15) and Bmi1 all show a very broad expression pattern in the corpus epithelial cells. The color bar indicates log2-transformed normalized read counts.

Methods S1: Punctuated neutral drift model, related to STAR Methods.

The theoretical model describing the clonal dynamics of labeled cells is based on an approach developed to determine stem cell dynamics in intestinal crypts (Lopez-Garcia et al., 2010; Snippert et al., 2010), which share major geometric features with stomach glands. In this framework, stem cells at the base of the crypt are represented by angular segments. Each of these angular segments represents a small angular region whose proliferative dynamics are governed by a single stem cell. Loss-and-replacement of a stem cell by a dividing adjacent stem cell occurs stochastically with a defined average rate λ and is represented by a segment being replaced with a copy of the neighboring segment (Figure 4A, upper panel). To describe the clonal dynamics observed in the multi-color Confetti mouse system, each segment may carry a color label representing a fluorescent marker gene. Since each segment has the same competitive potential, these cell fate dynamics lead to "neutral competition" between clones. Here we adapt this approach to describe lateral clonal expansion close to the vertical center of the isthmus region.

As described in the main text, we extend the above model by explicitly including barrier segments that account for the presence of parietal cells (Figure 4A, lower panel)—this allows us to compare experimental scenarios with and without the depletion of parietal cells and to draw conclusions about their role in limiting the rate of lateral stem cell loss-and-replacement. These barrier segments block lateral expansion of clones, so that only when a barrier segment is lost does a time window open for lateral expansion across its former position. The basis of this model assumption relies on a key observation: in the vast majority of cases, clones contain parietal cells bearing the same confetti color after 6 m post-labelling. More precisely, when we checked 73 clones (from n=3 mice) with a circumferential span of more than 180° from the 6 m tracing data using *Rosa26-CreERT2;R26R-Confetti* mice, all of these clones contained parietal cells bearing the same confetti color as neighboring cells (Figure S3E), implying that newly-generated parietal cells originate from neighboring IsthSCs. As we seldom observe clones containing different colored parietal cell(s) in the middle of the clones in the long-term, we believe that the crossing events where clone expansion occurs by "skipping over" the live parietal cell barrier are largely inhibited unless a parietal cell is lost.

To estimate the number and spatial distribution of parietal cells, we used experimental images of cross-sections of Confetti-labeled glands in the isthmus region. We then generated statistics of the numbers and relative angular positions of the parietal cells in the glands, both at 2 w and 3 m post-labeling. Counting the number of parietal cells across a large number of glands revealed a characteristic distribution, with most glands comprising 2 parietal cells, independent of the time point analyzed (dark blue bars in Figure 4B). As detailed in the main text, parietal cells are not randomly distributed in the glands, but instead seem to maximize the distance between neighbors (Figure S3F and dark blue bars in Figure 4C and S3G). In our model, we take these observations into account by a position-dependent probability for the birth of new barrier segments that depends on the location of already existing barriers, as described below.

Model description

In the current model, the state of the system is characterized by (i) the color label of *N* segments representing IsthSCs, and (ii) the presence or absence of barrier segments and, if present, their color label (Figure 4A, lower panel). Each barrier segment has an equivalent size of s_{B} stem cell-associated segments. All loss-and-replacement processes of stem cell-associated and barrier segments are Poisson processes with transition rates specified below. Loss-and-replacement of a stem cell-

associated segment occurs with a transition rate λ and amounts to a segment being replaced by a copy of one of its randomly selected neighboring segments, thus inheriting its color label. Barrier segments are sporadically born between proliferating segments. The birth rate at a certain position depends on the location of already existing barriers such that barrier segments tend to be localized with a characteristic distance between each other. Labeling the positions between stem cell-associated segments by the index *i*, the transition rate for a barrier segment to appear at a position *i* is given by

$$\Omega_i = \omega_{\text{in}} \prod_j \phi(n_{ij}) \,, \tag{1}$$

where ω_{in} is the reference birth rate for barrier segments, *j* runs over the indices of all currently present barrier segments, n_{ij} is the number of segments between position *i* and barrier segment *j* (which also depends on other potentially present barrier cells between the two positions), and the function ϕ determines how the birth rate depends on the distance n_{ij}. To reflect the fact that barrier segments are preferentially located far away from each other, ϕ should vanish for small distances and should be close to 1 for angular distances close to 180°. A generic function that satisfies these requirements and which we choose here is the Gaussian function

$$\phi(n) = \mathrm{e}^{-\frac{(n-N/2)^2}{2\sigma^2}} \,, \tag{2}$$

where σ is the characteristic length scale (measured in segments) over which the probability drops to zero outside an existing barrier segment, and $N/2$ is the maximum distance between segments in the absence of any barrier. When born, barrier segments randomly attain the color label of one of their neighboring stem cell-associated segments and, once present, the loss of barrier segments occurs with a rate ω_{out}. Only when a barrier segment is absent can its two neighboring stem cell-associated segments undergo mutual loss-and-replacement (Figure 4A). This fully characterizes our model of lateral expansion in the isthmus region. Stochastic realizations of the system were computed and statistics performed on the model results to obtain clone size distributions and the number and angle distribution of barrier segments using a standard stochastic simulation algorithm (Gillespie, 1977).

Simulating control and DMP-777 experiments

We used this model to simulate both control and DMP-777 experiments. To simulate control experiments, segments were color-labeled with barrier segments fully equilibrated, i.e. with their steady state number and angle distribution. Each segment attained a color label with an induction probability p_{ind}, which is another model parameter. To simulate DMP-777 experiments, segments were color-labeled in the absence of any barrier segment; during the course of the simulation, the barrier segment distribution then reaches its steady state in parallel with the ongoing clonal dynamics. Apart from the initial conditions, all parameter values are taken to be the same in control and DMP-777 experiments.

Determining a parameter set

To compare our model with experimental data, we obtained a reference parameter set in two steps. First, we independently constrained parameters that dynamically govern the number and angle distribution of barrier segments. We then focused on the remaining parameters that mainly affect the lateral clonal expansion dynamics.

We estimated the typical width of a parietal cell from experimental images as an equivalent of $s_{\text{B}} = 2$ stem cell-associated segments. The steady-state number and angle distribution of barrier segments are determined by the potential width σ and the ratio $r = \omega_{\text{out}}/\omega_{\text{in}}$ of loss rate and reference birth

rate of barrier segments. To constrain these two parameters, we compared the experimentally determined number distribution $P_{\mathrm{B}}^{\mathrm{exp}}(n)$ of parietal cells with the theoretical steady-state probability $P_{\mathrm{B}}^{\mathrm{theor}}(n|\sigma, r)$ to find *n* barrier segments in a gland given the parameters σ and *r*. No information about the angle distribution was used to inform the analysis. Following a standard least-squares approach, we then determined the residual $d(\sigma, r) = \sum_n (P_{\mathrm{B}}^{\mathrm{exp}}(n) - P_{\mathrm{B}}^{\mathrm{theor}}(n|\sigma, r))^2$ between these two distributions (Figure S3H). Subsequently, we restricted parameter combinations (σ, r) to regions in which the residual d was not larger than the 70-fold minimum of d (Figure S3H), which ensured that the region did not exceed the upper parameter boundaries.

Having found an independent way to constrain two parameters, we determined the remaining parameters, i.e. the loss-and-replacement rates λ, ω_{in} and the induction frequency p_{ind} (together with σ and *r* in the considered region) by a fit to average clonal properties. To strengthen the predictive value of the model, we used only seven data points characterizing the time-dependent average clone size for both control and DMP-777 experiments (Figure 4D and 4F) to fit these parameters. In addition to the best fit parameters, we constructed a 95% CI for each parameter using a basic bootstrap method, which consisted of resampling from experimental clone size distributions and refitting the model to the resampled population so that a distribution of fit parameters is obtained (Roff, 2006). The confidence bounds were then defined as the 2.5%- and the 97.5%-quantile of the parameter distribution. The corresponding fit values and confidence bounds are given in Table S1.

Comparison with experiments

Using the obtained parameter set as a reference, we then assessed whether the model could capture the experimental data. Considering the barrier segments first, we found (i) that the model fit (sky blue dots and lines in Figure 4B) converged to a good agreement with the observed number distribution and (ii) also yielded a good quantitative prediction for the angle distribution conditioned on the number of barrier segments in the glands (sky blue dots and lines in Figure 4C and S3G). Turning to the clone size distribution, we found that the model fit could capture quantitatively the average clone size under both control and DMP-777 conditions using the same parameter set and only different initial conditions, as described above. Moreover, the model also captured the overall features of the full clone size distribution (Figure 4E, 4G and S3J) and the number of labeled glands as a function of time (Figure S3K).

Table S1: Parameter set for the lateral expansion model, related to Figure 4 and Methods S1.

Param.	Units	Value	Confidence bounds	Description
N		8		number of stem cell-governed segments
λ/N^2	1/month	0.3	–0.03 / +0.13	loss-and-replacement rate of stem cell-governed segments
ω_{in}	1/month	0.4	–0.13 / +0.13	reference birth rate of barrier segments
ω_{out}	1/month	0.25	–0.08 / +0.05	loss rate of barrier segments
σ	segments	0.3	–0.1 / +0.1	"potential width" for birth of barrier segments (see text)
s_B	segments	2		equivalent width of barrier segments
n_C		2		number of color labels
p_{ind}		14 %	–2% / +4%	induction frequency
